# Scale-insensitive estimation of speed and distance traveled from animal tracking data

**DOI:** 10.1186/s40462-019-0177-1

**Published:** 2019-11-15

**Authors:** Michael J. Noonan, Christen H. Fleming, Thomas S. Akre, Jonathan Drescher-Lehman, Eliezer Gurarie, Autumn-Lynn Harrison, Roland Kays, Justin M. Calabrese

**Affiliations:** 1 Smithsonian Conservation Biology Institute, National Zoological Park, 1500 Remount Rd, Front Royal, 22630 USA; 2 Department of Biology, University of Maryland, College Park, 20742 USA; 3 Department of Biology, George Mason University, 4400 University Drive, Fairfax, 22030 USA; 4 Migratory Bird Center, Smithsonian Conservation Biology Institute, National Zoological Park, Washington, DC, 20008 USA; 5 North Carolina Museum of Natural Sciences, Biodiversity Lab, Raleigh, 27601 USA; 6 Department of Forestry & Environmental Resources, North Carolina State University, 4400 University Drive, Raleigh, 27695 USA

**Keywords:** Continuous-time, correlated velocity, ctmm, GPS, movement models, step length, telemetry, travel distance

## Abstract

**Background:**

Speed and distance traveled provide quantifiable links between behavior and energetics, and are among the metrics most routinely estimated from animal tracking data. Researchers typically sum over the straight-line displacements (SLDs) between sampled locations to quantify distance traveled, while speed is estimated by dividing these displacements by time. Problematically, this approach is highly sensitive to the measurement scale, with biases subject to the sampling frequency, the tortuosity of the animal’s movement, and the amount of measurement error. Compounding the issue of scale-sensitivity, SLD estimates do not come equipped with confidence intervals to quantify their uncertainty.

**Methods:**

To overcome the limitations of SLD estimation, we outline a continuous-time speed and distance (CTSD) estimation method. An inherent property of working in continuous-time is the ability to separate the underlying continuous-time movement process from the discrete-time sampling process, making these models less sensitive to the sampling schedule when estimating parameters. The first step of CTSD is to estimate the device’s error parameters to calibrate the measurement error. Once the errors have been calibrated, model selection techniques are employed to identify the best fit continuous-time movement model for the data. A simulation-based approach is then employed to sample from the distribution of trajectories conditional on the data, from which the mean speed estimate and its confidence intervals can be extracted.

**Results:**

Using simulated data, we demonstrate how CTSD provides accurate, scale-insensitive estimates with reliable confidence intervals. When applied to empirical GPS data, we found that SLD estimates varied substantially with sampling frequency, whereas CTSD provided relatively consistent estimates, with often dramatic improvements over SLD.

**Conclusions:**

The methods described in this study allow for the computationally efficient, scale-insensitive estimation of speed and distance traveled, without biases due to the sampling frequency, the tortuosity of the animal’s movement, or the amount of measurement error. In addition to being robust to the sampling schedule, the point estimates come equipped with confidence intervals, permitting formal statistical inference. All the methods developed in this study are now freely available in the ctmmR package or the ctmmweb point-and-click web based graphical user interface.

## Background

Understanding how far animals must travel to meet their nutritional and/or reproductive requirements, as well as the rate at which these distances are covered, are fundamental components of ecological research [1, 2]. Collectively, speed- and distance-related movement metrics provide quantifiable links between behavior and energetics [1, 3–6], can inform on risk/reward tradeoffs (*sensu* Charnov [7]), and can be important signals for the extent of anthropogenic disturbance [8, 9]. Accurately quantifying variations in an animal’s movement speed over time can also enable explorations into the behavioral mechanisms animals use to navigate their environment [10]. For instance, when individuals exhibit area restricted search (*sensu* Kareiva [11]), they are expected to slow down and move more tortuously in areas of high resource density, and speed up and move more ballistically in areas of low resource density (see also [12]).

Animal tracking data are becoming an increasingly important resource for addressing these questions [13], with distance traveled typically being quantified by summing the straight-line displacement (SLD) between discretely sampled locations [14–17]. Similarly, dividing this value by the time elapsed between location observations is used to estimate an animal’s speed (but see the instantaneous-speed estimation method of Johnson et al. [18], and the Gaussian, mean-speed estimation methods of Calabrese et al. [19], and Gurarie et al. [20]). Although straightforward to calculate, approximating a non-linear movement path by a series of linear segments has long been known to underestimate the true distance traveled at coarse sampling frequencies [12, 14–17, 21, 22]. All else being equal, the extent of this bias will tend to increase with both the amount of tortuosity in the animal’s movement and the coarseness of the sampling [16]. As a correction to this scale-sensitivity, it is suggested that increasing the sampling frequency will improve the accuracy of SLD estimates, as linear segments of smaller lengths more accurately capture the shape of non-linear paths [16]. Problematically however, animal tracking data are also subject to measurement error [23, 24]. When paths are sampled at fine temporal scales, measurement error becomes a major source of bias and SLD will tend to over-estimate the true distance traveled [25]. To see this, consider an individual tracked at a one-minute sampling interval. If, during that interval, it travels an average of 5m, but the measurement error on each location is 10m, the error will be larger than the scale of the movement, and will dominate the estimated distance traveled. The suggested approach to correct for error induced bias is to smooth the data by fitting a movement model to the data to jointly estimate measurement and process variances, and then apply SLD on the smoothed data [26, 27]. However, the fundamental limitations with this type of approach are that joint estimation has serious identifiability issues [28] which can lead to under- or over-smoothing, while coarse-scale tortuosity induced bias is still not accounted for. Compounding the issue of the sensitivity of SLD estimation, these estimates do not come equipped with confidence intervals to quantify their uncertainty. This means that it is not currently possible to determine if a set of SLD-based estimates are statistically different from one another. These issues present serious problems for any comparative analyses because SLD estimates are not only influenced by how far the animal traveled, but also by the sampling frequency [14, 15, 22], the tortuosity of the animal’s movement [16], and the amount of measurement error [25].

Importantly, the continuous nature of animal movement means that as individuals navigate through their environment their positions and, crucially in the context of speed/distance estimation, velocities are necessarily autocorrelated over time [20]. Here, we take advantage of these fundamental properties of motion to overcome the scale-sensitivity of SLD estimation. We outline how to estimate speed, both average and instantaneous, and distance traveled in a scale-insensitive way that builds upon the existing continuous-time movement modeling framework [18, 19, 29–33]. Modeling movement in this framework separates the continuous-time structure of the underlying movement process from the discrete-time structure of the sampling process [29, 34–36], which allows for inference that is less sensitive to the sampling schedule than discrete-time approaches [37]. Our approach makes use of the error [29, 32], and correlated velocity components of these models [20, 29] to estimate speed and distance traveled as latent variables (i.e., indirectly observed variables that are inferred from directly observed variables). Crucially, not only does this approach allow for scale-insensitive estimation of these movement metrics, but it also provides a means of obtaining confidence intervals. We first use a series of simulations to demonstrate the influence of each source of bias on SLD estimation (i.e., sampling frequency; random data loss; tortuosity; and measurement error). We then use a similar set of simulations to show how the continuous-time approach we detail can correct for these sources of bias and provide accurate estimates. Finally, we demonstrate the utility of our approach, and the sometimes radical improvements it can provide versus both conventional and model-smoothed SLD, on GPS data from a wood turtle (*Glyptemys insculpta*) tracked in Virginia, USA, and a white-nosed coati (*Nasua narica*) tracked on Barro Colorado Island, Panama.

## Methods

### Universal data limitations for speed/distance estimation

A currently unrecognized aspect of speed/distance estimation is that, irrespective of what estimator is applied to the data, this analysis is not necessarily appropriate for every dataset. We therefore begin by detailing this limitation so as to place the work that follows in its proper context.

An animal’s true location in two dimensions, **r**(*t*), at time *t* is defined by the location vector

1 $$\begin{array}{*{20}l} \mathbf{r}(t) = (x(t),y(t)) \,. \end{array} $$

While an animal’s *displacement* over a certain timeframe, (*t*_1_,*t*_2_), is the straight line displacement between true locations **r**(*t*_1_) and **r**(*t*_2_), the *distance* that it traveled, *d*(*t*_1_,*t*_2_), is the integral of its speed, *v*(*t*), with respect to time

2 $$\begin{array}{*{20}l} d(t_{1},t_{2}) = \int_{t_{1}}^{t_{2}} \!\!\! v(t) \, dt \,, \end{array} $$

where speed is the magnitude of the velocity vector, **v**(*t*), given by

3 $$\begin{array}{*{20}l} v(t) &= | \mathbf{v}(t) | = \sqrt{ v_{x}(t)^{2} + v_{y}(t)^{2}} \,. \end{array} $$

Finally, for any given time, an animal’s velocity is the derivative of its true position with respect to time,

4 $$\begin{array}{*{20}l} \mathbf{v}(t) &= \frac{d}{dt} \mathbf{r}(t) \,. \end{array} $$

From these fundamental relationships, we see that estimating speed and/or distance traveled from location data requires that there be information on velocity in the data. Conversely, if no velocity information exists, then speed/distance estimation is inappropriate, irrespective of what estimator is used.

**Fig. 1:**
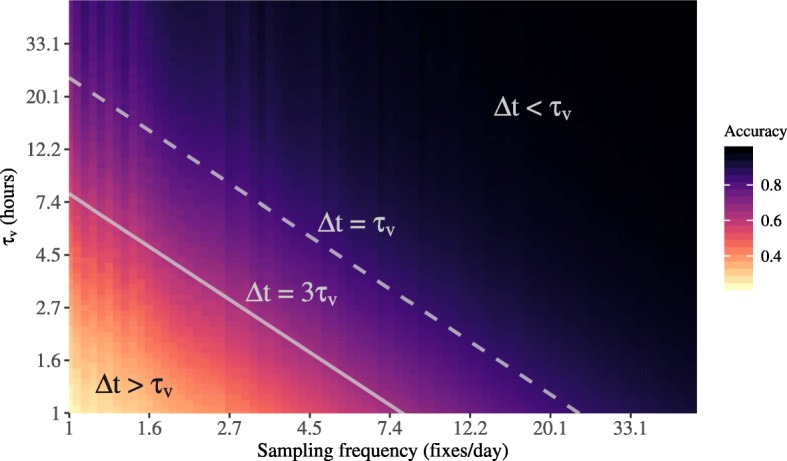
The results of simulations demonstrating the inability to obtain an accurate estimate via straight line displacement (SLD) when the sampling interval, *Δt*, is longer the the velocity autocorrelation timescale, *τ*_*v*_, and the severe bias when *Δt*≥3*τ*_*v*_. For details on the simulations, see Additional file 1

As noted above, the continuous nature of animal movement means that positions and velocities are necessarily autocorrelated over time [20, 38]. Animals with strong directional persistence (e.g., as in a migratory individual), will tend to have long velocity autocorrelation timescales, *τ*_*v*_. Animals with more tortuous movement in contrast, will tend to have a much shorter *τ*_*v*_. The relationship between *τ*_*v*_ and the sampling interval, *Δt*, is, therefore, critical for determining whether there will be any signature of the animal’s velocity, and hence movement path, in the data. More specifically, because velocity autocorrelation decays exponentially at rate 1/*τ*_*v*_, the time required for the proportion of the original velocity autocorrelation to decay to *α* is *τ*_*α*_=*τ*_*v*_ ln(1/*α*). Conventionally 5% or less autocorrelation remaining in the data is considered effectively independent, so ∼3*τ*_*v*_ is the time it takes for 95% of the velocity autocorrelation to decay. Therefore, if *Δt*>3*τ*_*v*_, no statistically significant signature of the animal’s velocity will remain in the location data, leaving insufficient information for accurate speed or distance estimation (Fig. 1). This means that such a dataset is simply too coarsely sampled to support speed/distance estimation, and this limitation applies regardless of which estimator is used. Further mathematical proofs on this universal data limitation are provided in Additional file 1.

### Bias in straight-line displacement (SLD) estimation

Animal tracking data are obtained by discretely sampling an animal’s location, **r**, at times *t*_*i*_∈{*t*_1_,…,*t*_*n*_}. From these data, distance traveled is typically quantified by summing the SLD between locations

5 $$\begin{array}{*{20}l} \hat{d} = |\Delta \mathbf{r} | &= \sqrt{\Delta x^{2} + \Delta y^{2}}. \end{array} $$

Further dividing this estimate by the change in time over which the movement occurred is used to estimate speed

6 $$\begin{array}{*{20}l} \hat{v} = \frac{\hat{d}}{\Delta t}. \end{array} $$

**Fig. 2:**
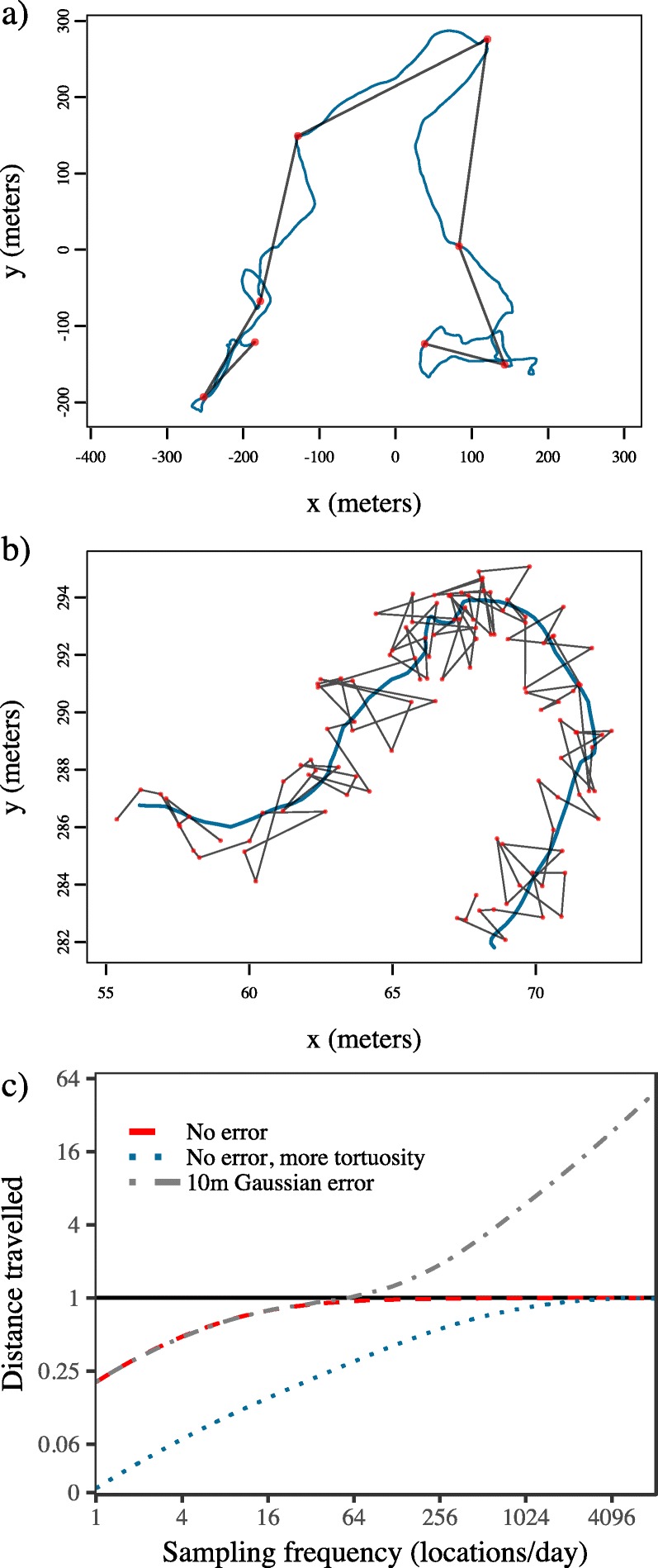
Examples of the sources of bias in straight line displacement (SLD) estimation for (**a**) coarsely sampled data that fail to capture the tortuosity of the animal’s movement; and (**b**) finely sampled data that are subject to measurement error. In both panels the blue line depicts the path the simulated animal actually traveled, the red dots the sampled locations, and the black lines the straight line displacements between locations. Note how SLD using the coarsely sampled data misses movement the animal actually made, whereas SLD using the finely sampled data introduces movement the animal did not make. In panel **c**, the results of simulations depict the trade-off of these sources of bias across scales. The solid black line depicts the true value to which the estimates should converge (scaled to 1), and both axes are log scaled. Movement paths were simulated from Ornstein-Uhlenbeck Foraging (OUF) processes. For the simulations depicted by the red and gray curves, the velocity autocorrelation timescale (*τ*_*v*_) was set to 1 h. For the blue curve, *τ*_*v*_ was set to 1 min, which produced more tortuous movement

Problematically, measuring the length of a non-linear movement path by summing a series of linear segments between true locations will always underestimate the true distance traveled unless the focal animal actually moved in perfectly straight lines between observations (Fig. 2a). This happens because discretely sampled tracking data represents only a subset of the animal’s full path, and the shortest distance between two points is a straight line. All else being equal, the extent of this bias will also be greater for individuals with more tortuous movement (see the blue, dotted line in Fig. 2c; see also [16]). Increasing the sampling frequency is often suggested as way of reducing this negative bias [14–16, 22], since decreasing the time between successive relocations results in shorter segments that better approximate the non-linear shape of the movement path — effectively functioning as a Riemann sum approximation of the path length [39].

Crucially, this approach is only valid if the true positions are known exactly (i.e., the red, dashed line in Fig. 2c). In reality however, the true positions are not known, as there is generally some extent of measurement error on the observations [23, 24]. If these errors are uncorrelated in time, SLD estimates actually diverge to infinity as the sampling frequency increases

7 $$\begin{array}{*{20}l} {\lim}_{\Delta t \to 0} \left|\frac{\Delta}{\Delta t} \underbrace{(\mathbf{r} + \mathbf{error})}_{\text{observable}}\right| = \infty. \end{array} $$

This happens because the actual distance traveled by the animal goes to 0 in the limit where *Δt*→0, but the magnitude of uncorrelated measurement error is independent of *Δt* (e.g., Fig. 2b). As a result, at short sampling intervals, the estimate becomes dominated by measurement error (see the gray, dashed line in Fig. 2c; see also [25]). Jointly estimating the movement and error variances, and then smoothing the data conditional on these fitted models has been suggested as a means of correcting for error induced bias [26, 27]. However, this type of approach is limited by the serious identifiability issues of joint estimation [28] which can lead to under- or over-smoothing of the data, while the coarse-scale, tortuosity induced bias is still not accounted for.

Collectively, this scale-sensitivity means that when animals are tracked at coarse temporal scales SLD will tend to underestimate their speed and distance traveled, yet will tend to overestimate these quantities when tracked at fine temporal scales. While, in principle, it is possible to adjust the sampling frequency such that these sources of bias cancel out, this would require knowing the error magnitude of the deployed tracking device and tortuousity in the animal’s movement *a priori*. Furthermore, tortuousity might vary substantially from one individual to the next [40] even within the same species tracked in the same place, at the same time [16], and measurement error can vary between tracking devices. In practice therefore, it would be extremely difficult to reliably hit this ‘Goldilocks’ sampling frequency, and missing it would mean biasing the results in one direction or the other. Using the sampling frequency to strike a balance between these sources of bias is thus an unreliable way of accounting for the scale-sensitivity of SLD estimation.

### Continuous-time estimation of speed/distance traveled

**Fig. 3:**
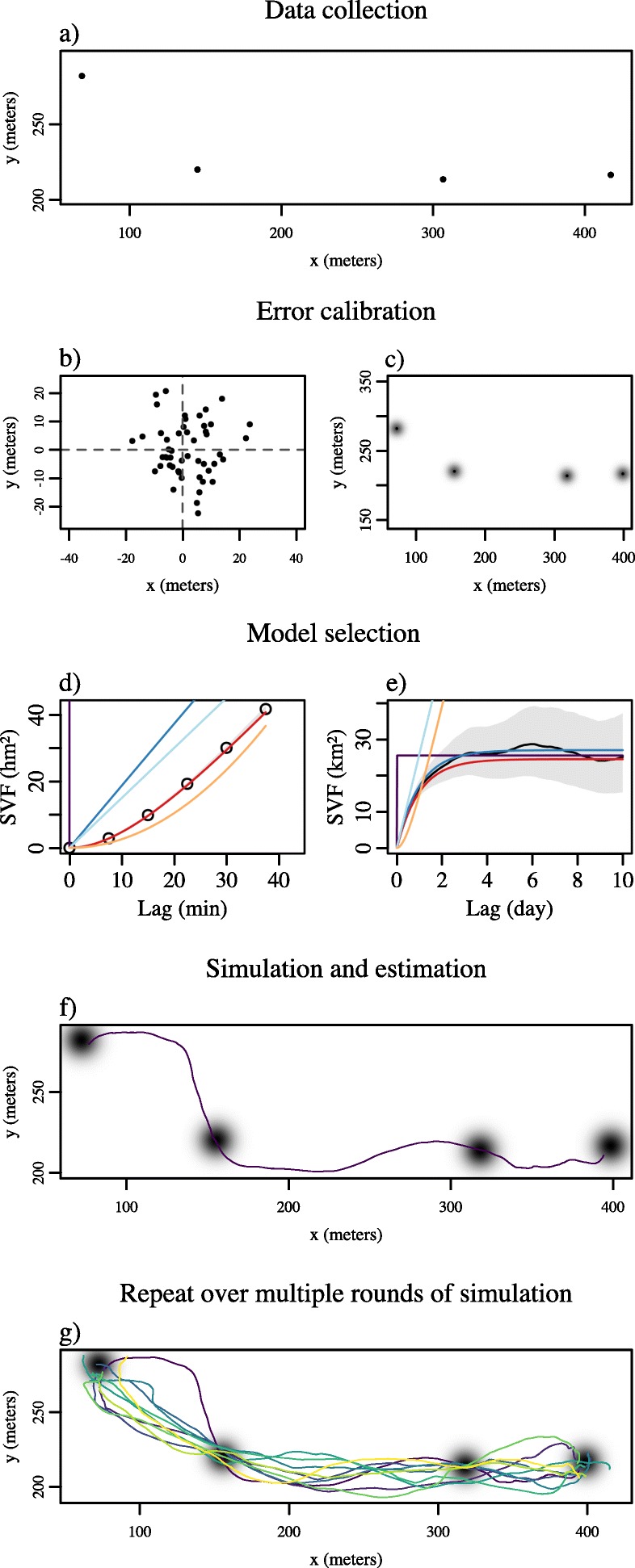
A walkthrough of the steps involved in our continuous-time speed and distance estimation (CTSD) method using simulated data. Beginning with the tracking data (panel **a**; here with a 1-hr sampling interval), the first step is to use some calibration data (panel **b**) to estimate the device’s RMS user equivalent range error (UERE). Once the errors have been calibrated (panel **c**), model selection techniques are employed to identify the best fit model for the fine-scale (panel **d**) and coarse-scale (panel **e**) features of the data — SVF represents the semi-variance function. A trajectory is then simulated, conditional on the data, the fitted movement model, and the calibrated error model (panel **f**), and the distance/speed of that trajectory is calculated. The simulated animal had a velocity autocorrelation timescale of 25 min, so the trajectory in panel **f** is simulated at a frequency of 2.5 min. The simulation and estimation step is then repeated over multiple rounds of simulation (panel **g**), and the ensemble provides a point estimate and 95% confidence intervals

To alleviate the scale-sensitivity of SLD estimation, we outline a scale-insensitive, continuous-time speed and distance estimation (CTSD) method that builds upon the existing continuous-time movement modeling framework [18, 19, 29–31, 33]. As described above, an inherent property of working in continuous-time is the ability to separate the underlying continuous-time movement process from the discrete-time sampling process. Consequently, continuous-time models are less sensitive to the sampling schedule when estimating parameters. Starting with some tracking data (Fig. 3a), the first step in our approach is to account for error in the position measurements [29, 32, 41]. This is done by using calibration data, where the tracking device has been left in a fixed location for a period of time (Fig. 3b), to estimate the device’s root mean square (RMS) user equivalent range error (UERE). RMS UERE is the device specific error, in meters, defined by the sum of errors resulting from receiver noise, satellite clocks, and tropospheric/ionospheric effects, given ideal satellite coverage [42]. For GPS data, the device specific RMS UERE is then used as a proportionality constant to translate the unit-less location specific errors, recorded in GPS dilution of precision (DOP) values (both horizontal, HDOP, and vertical VDOP), into standard deviations of mean-zero error (Fig. 3c), where the location error=RMS UERE×HDOP [43]. Assuming functional devices, RMS UERE values should apply to all tags of a given type, while DOP values capture the large location-to-location differences in measurement error. Note, ARGOS data [44], and some brands of GPS tracking devices come pre-calibrated. In such cases, the additional step of collecting calibration data to transform the DOP values is not necessary. To calibrate the errors we used the uere.fit() function from the ctmm package (Fleming et al. Getting a handle on telemetry error, in preparation). After data import and error calibration, we recommend that the data be inspected for outlying data points, and all outliers should be removed prior to analysis (for examples of this process see Additional file 2.

The next step is to fit a continuous-time, correlated-velocity movement model that appropriately describes the animal movement data. As noted above, speed and distance traveled are properties of an animal’s velocity over time, and the capacity to estimate these quantities is linked to the ability to resolve *τ*_*v*_. If the data are too coarsely sampled, relative to the animal’s movement, to be able to fit a correlated velocity model [20], it will not be possible to estimate speed/distance, as the data will no longer contain any signature of the path the animal traveled between locations (see also Additional file 1). Here, it is also important to fit the error and movement models separately because, if fit simultaneously, it can be difficult for the models to distinguish between actual movement and error, and parameters can be confounded [28]. This second step, therefore, begins by holding the error model fixed after calibration, and then employing model selection techniques to identify the best continuous-time movement process for the data [36, 38]. Models are fit using perturbative hybrid residual maximum likelihood (pHREML; [45]), and the best movement model for the data selected using small-sample-size corrected Akaike’s Information Criterion (AICc; [19]), using the R package ctmm, applying the workflow described by [19]. Notably, if model selection favors a model without correlated velocities, such as OU motion [46], or Brownian Motion [47], this is an indication that the data are too coarsely sampled to support velocity estimation. The selection of a correlated velocity process, such as Integrated Ornstein-Uhlenbeck (IOU) motion [29] or Ornstein-Uhlenbeck Foraging (OUF) motion [30], is necessary to proceed to the next steps of speed and distance estimation (Fig. 3d, e). To fit and select the movement, and error models, we use the R package ctmm, applying the workflow described by [19], which includes all stationary, continuous time-models currently in use in the ecological literature [32]. Although these models return immediate Gaussian estimates of the RMS speed [19, 20] (detailed in Additional file 3), RMS speed is not necessarily proportional to the total distance traveled, and the true velocities, **v**(*t*), are not necessarily normally distributed. Obtaining a non-parametric estimate of speed, whose time average is proportional to distance traveled, requires an additional simulation step that we describe here.

Once appropriate error and movement models have been estimated, the final step is to simulate a series of error-free trajectories conditioned on the data, with a sampling interval that is much smaller than the velocity autocorrelation timescales (Fig. 3f). At scales much shorter than the velocity autocorrelation timescales, the instantaneous velocities become approximately constant over short time intervals, and the simulated data are therefore more appropriate for straight-line interpolation. When calculating mean speeds and distances, numerical errors from this discretization are $\mathcal {O}(\Delta t^{3})$, with shorter intervals (*Δt*) producing more accurate estimates. The computation time, however, scales inversely with *Δt*, where shorter intervals increase the computation time. Consequently, there is a trade-off between accuracy and computation time, and we chose $\Delta t = \frac {\tau _{v}}{10}$, where *τ*_*v*_ is the velocity autocorrelation timescale, which has a corresponding relative error of $\mathcal {O}(10^{-3})$. In terms of the number of simulated trajectories, our approach first simulates 20 trajectories and then continues to batch simulate trajectories until the standard error reaches the target error threshold (here 10^−3^). For each of these simulated trajectories, we calculate the instantaneous speeds

8 $$\begin{array}{*{20}l} v(t_{i}) = \sqrt{v_{x}(t_{i})^{2} + v_{y}(t_{i})^{2}}, \end{array} $$

and use these to estimate total distance traveled (*d*), and average speed ($\bar {v}$) using the trapezoidal rule (i.e., the average of the left and right Riemann sums; [39])^1^

9 $$\begin{array}{*{20}l} d &= \sum_{i}(\Delta t_{i} |v(t_{i}) |)&\bar{v} &= \frac{\sum_{i}(\Delta t_{i} |v(t_{i})|)}{\sum_{j} (\Delta t_{j})}. \end{array} $$

Repeating this third step over multiple rounds of simulations (Fig. 3g) provides an ensemble of estimates from which the mean speed, $\langle \bar {v} \rangle $, and/or distance 〈*d*〉 can be estimated. Because this method relies on generating an ensemble of values that are influenced by process, measurement, and parameter uncertainty, it is also possible to calculate the variance around the point estimate as well as confidence intervals. The estimates range on a scale from 0 to infinity, so as an improvement over normal CIs, which can include negative values, we summarize the uncertainty of this ensemble with *χ* statistics. These are exact for the mean speed of a stationary Gaussian process with isotropic variance, as its location (and derivatives thereof) are normally distributed with equal variance in every direction (see Additional file 3).

The methods we describe here are fully implemented in the R package ctmm (version 0.5.7 and higher), as well as in the point-and-click web based graphical user interface at ctmm.shinyapps.io/ctmmweb/ (version 0.2.5; [48]). Average speed or distance traveled can be estimated via the speed() function, whereas instantaneous speeds can be estimated using the speeds() function. While this workflow involves several steps, the ctmm R package and ctmmweb point-and-click web based graphical user interface streamline this procedure, and full examples of the workflow are shown in Additional file 2.

### Simulation study

We first used simulated data to explore how the bias of SLD estimation, both conventional and model-smoothed, as well as CTSD, varied with sampling frequency, movement tortuosity, random data loss, and measurement error. Although CTSD permits estimation of both instantaneous and mean speed, as well as total distance travelled, for conciseness we only evaluated the distance traveled estimates in our simulation study, as these are the most directly related to the conventional SLD estimates. Data were simulated based on an OUF process, which features a home range, correlated positions, and correlated velocities (for full details on this model see [30]). The OUF process is representative of modern GPS tracking data commonly used in these analyses [49], and tends to apply frequently in practice [40]. Data were simulated according to four sets of manipulations:

1. **Sampling frequency**. In our first set of simulations, we tested how variation in sampling frequencies influenced estimates. We set the position and velocity autocorrelation timescales to 1 day, and 1 h respectively, which are typical timescales for these parameters in many medium-sized, range-resident mammals [19, 36, 50]. From this model, we simulated a fine scale trajectory, sampled for 10 days at a frequency of 4096 locations/day. This fine-scale, error-free trajectory was used to estimate the true distance traveled — for small time steps the Riemann sum converges to the truth. After determining the truth, mean-zero Gaussian error with a standard deviation of 10m was added to each location. Using the data with error, we estimated the total distance traveled using both conventional SLD and CTSD estimation. Further to conventional SLD, we also estimated model-smoothed SLD *sensu* [26, 27]. For this latter approach, we applied the standard ctmm workflow [19, 51] to jointly estimated the process and error variances sans calibration data. We then used the estimated movement and error models to smooth the data by predicting the most likely location at each of the sampled times. Finally, we calculated SLD estimates on these smoothed data. We note that because all of the simulated data were generated from stationary, OUF processes, the true model was within the set of candidate models. So this was a best case scenario for how model-smoothed SLD can be expected to perform in practice. We then compared these three estimates to the truth. We next thinned down the fine-scale trajectory by removing every second location, and repeated the model fitting and estimation process. This thinning and re-estimation was repeated to generate increasingly coarse data with sampling frequencies that ranged from the full resolution of 4096 locations/day, down to 8 locations/day in a halving series. Fewer than 8 fixes per day resulted in an OU model being selected for this parameterization (i.e., with a velocity autocorrelation timescale of 1 h, a 3 h interval was where *Δt*=3*τ*_*v*_ and no statistically significant signature of the animal’s velocity remains in the data).
2. **Irregular sampling**. In our second set of simulations, we tested the performance of SLD and CTSD on data with irregular sampling, where we mimicked the effect of sporadic data loss, which is a common issue with tracking data [52], and known to present issues to discrete time methods [53, 54]. We set the position and velocity autocorrelation timescales to 1 day, and 1 h respectively, and simulated a trajectory sampled for 10 days at a constant frequency of 64 locations/day. Again, after determining the truth, mean-zero Gaussian error with a standard deviation of 10m was added to each location. We then randomly dropped a percentage of the collected locations (ranging from 0% — i.e., no data loss — to 70%, and increasing by 5% increments), where increasing the percentage of data loss resulted in increasingly irregular data. Using the irregularly thinned data with error, we estimated the total distance traveled using both conventional and model-smoothed SLD, as well as CTSD estimation, and compared these estimates to the truth.
3. **Movement tortuosity**. In our third set of simulations, we tested how variation in the tortuosity of an individual’s movement influenced estimates. Here, we simulated a trajectory sampled for 10 days at a constant frequency of 64 locations/day. We set the position autocorrelation timescales to 1 day, but manipulated the velocity autocorrelation timescale (ranging from 11.25 min to 1 day in a doubling series), where increasing the duration of velocity autocorrelation generates movement that is decreasingly tortuous (i.e., more linear, [30]). After determining the truth, mean-zero Gaussian error with a standard deviation of 10m was added to each location. The total distance traveled was then estimated using both conventional and model-smoothed SLD and CTSD as described above, and these estimates were compared to the truth.
4. **Location error**. In our fourth set of simulations, we tested how variation in the amount of measurement error influenced estimates. Here, we simulated 100 trajectories, sampled for 10 days at a fixed frequency of 64 locations/day. We set the position and velocity autocorrelation timescales to 1 day, and 1 h respectively, resulting in $\Delta t \approx \frac {1}{3} \tau _{v}$. After simulation, we again added mean-zero Gaussian error to each location, but here manipulated the standard deviation (ranging from 0, i.e., no error, to 51.2 meters, in a doubling series of the minimal value of 0.1 m error).

The simulations we described above were aimed at determining how CTSD, with a correctly calibrated error model, compared to SLD estimation. However, bias can still be introduced to the CTSD method if the error model is poorly specified. To evaluate the potential severity of this bias, we further compared CTSD distance traveled estimates for three different model fitting approaches; 1) fitting the movement model without error; 2) fitting the movement and error models simultaneously *sensu* [28]; and 3) fitting the movement and error models separately (i.e., the full approach described above). The parameterization of the simulation was identical to the sampling frequency simulation described above. The total distance traveled was then estimated using SLD and CTSD with the three error handling approaches, and these estimates were compared to the truth.

Each of these simulation studies was repeated 100 times, and we compared the mean performance of each estimator. All simulations were performed in the R environment (version 3.5.1; [55]) using the methods implemented in the R package ctmm (version 0.5.7; [19]), and the computations were conducted on the Smithsonian Institution High Performance Cluster (SI/HPC). The code necessary to reproduce these simulations is presented in Additional file 4.

### Empirical case studies

To verify that the estimators would, in practice, perform as they did on the simulated data, we tested both conventional and model-smoothed SLD, and CTSD on GPS relocation data for a wood turtle, and a white-nosed coati [56]. For the wood turtle, locations were sampled every hour over a 42 day period in autumn, 2016. Calibration data for this animal’s tracking tag were collected by leaving two devices of the same model in a fixed location for 1 day, and sampling at 10 min intervals. From these calibration data, the tracking device was found to have a horizontal RMS UERE of 10.6 meters, while the tracking data had a median HDOP of 1.4 (ranging from 0.8 – 9.9). For the white-nosed coati, which tend to exhibit very tortuous movement [57], locations were sampled every 15 min over a 41 day period in spring 2010, using e-obs collars with a median horizontal accuracy estimate of 15.6 meters (ranging from 2.6 – 78.3 meters). E-obs devices come pre-calibrated, so, for these data, no additional calibration was necessary.

We selected these datasets not because CTSD is restricted to terrestrial, GPS tracking data, but to highlight two general cases that are likely to occur in practice: i) the case where the movement and measurement error are on approximately the same scale, resulting in *a priori* unpredictable biases in SLD estimates (i.e., the white-nosed coati data); and ii) the case where the amount of measurement error is much larger than the amount of movement that occurs between positional fixes, resulting in positively biased SLD (i.e., the wood turtle data). However, in addition to these GPS examples, Additional file 2 provides a worked example of CTSD applied to ARGOS data from a brown pelican (*Pelecanus occidentalis*), tracked on the eastern coast of the United States.

For each of these datasets we first fit the full suite of movement models described above, and performed model selection to identify the most appropriate model for the data. We then estimated the total distance traveled using SLD, both conventional and model-smoothed, and CTSD. To evaluate the scale-sensitivity of these empirical estimates, we subsequently thinned the data by dropping every second location, and repeated the model fitting/selection, and distance estimation steps on these coarser data. This thinning and estimation process was repeated iteratively until the data became too coarse to be able to select a correlated-velocity model (i.e., *Δt*>3*τ*_*v*_). To further evaluate how SLD and CTSD estimates might compare in practice, we also estimated the daily distance traveled using SLD and CTSD, which is a routinely estimated metric.

## Results

### Simulation results

**Fig. 4:**
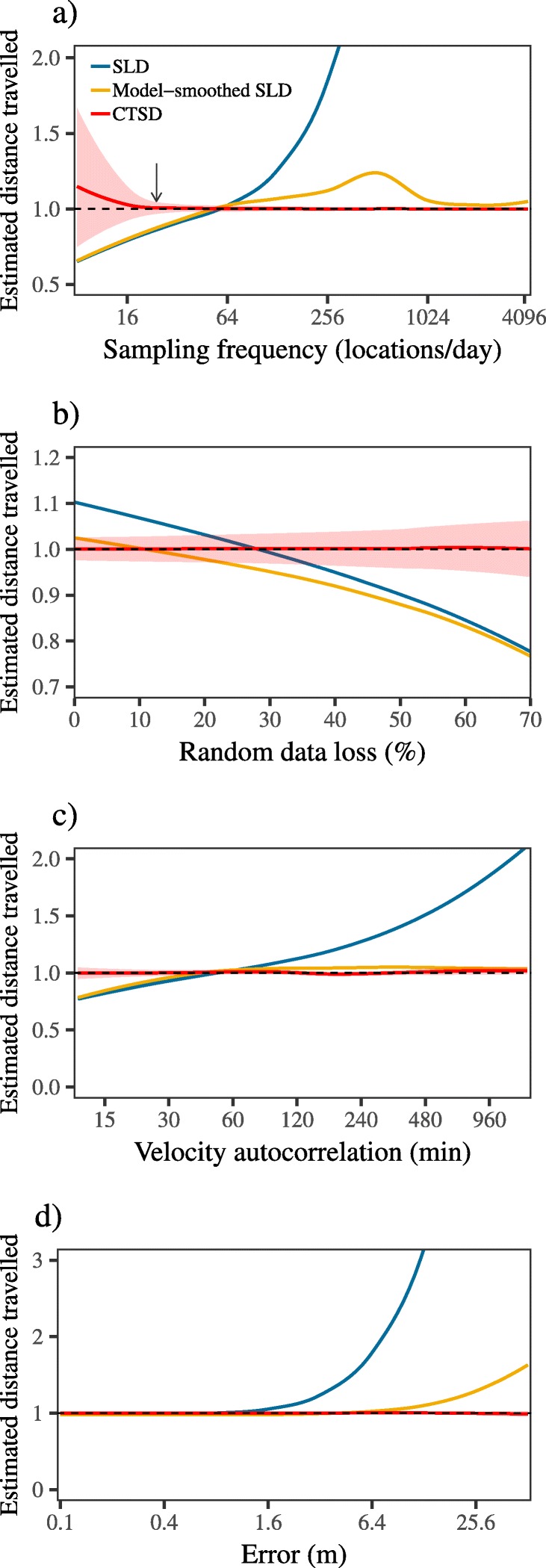
Figure depicting the results of simulations quantifying distance traveled via straight line displacement, and the continuous-time estimation method for manipulations of (**a**) sampling frequency; (**b**) the amount of random, irregular data loss; (**c**) the tortuosity of the underlying movement; and (**d**) the amount of measurement error. For the red line, the shaded area represents the 95% CIs (SLD estimates, both model-smoothed and conventional, do not come with CIs). The arrow in panel (**a**) depicts the point at which the sampling interval, *Δt*, is the same as the velocity autocorrelation timescale, *τ*_*v*_. In all panels, the dashed line at *y* = 1 depicts the true value to which the estimates should converge and the *x*-axis is log scaled. Note: the truth has been scaled to 1

**Fig. 5:**
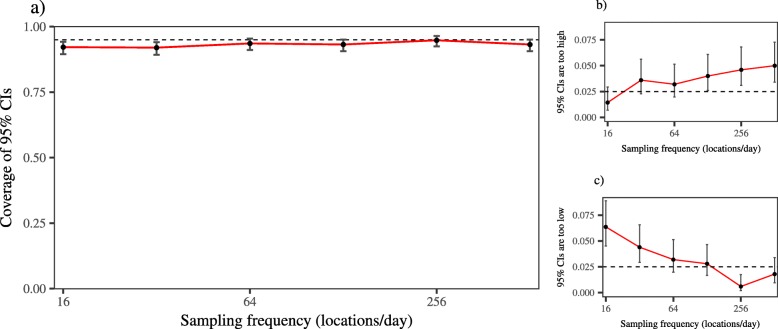
Figure depicting (**a**) the coverage of the 95% confidence intervals, as well as (**b**) the proportion of cases where the coverage of the confidence intervals was higher than, and did not include the true value; and (**c**) lower than, and did not include the true value. In all panels the error bars represent the 95% confidence intervals on the estimated coverage, the dashed line depicts nominal coverage, and the *x*-axis is log scaled

From these simulations, we found SLD estimates to be significantly biased by variation in sampling frequency, with substantial under-estimation at coarse resolutions, over-estimation at fine resolutions, and only a narrow window when $\sqrt { \frac { \text {VAR}[ \text {error} ] }{ \text {VAR}[ \text {velocity} ]} } \ll \Delta t \ll \tau _{v}$ where these contrasting sources of bias cancelled out to provide an accurate estimate (Fig. 4a). Model-smoothed SLD did provide some correction for error induced bias in SLD estimation for finely sampled data, but still resulted in negatively biased estimates for coarsely sampled data. In contrast, CTSD provided consistently accurate estimates across the majority of the sampling frequencies we examined, and was the only scale-insensitive estimator of those examined here. We note that when *Δt*>*τ*_*v*_, CTSD resulted in some positive bias. Despite this positive bias, we found that as the sampling became increasingly coarse, the 95% confidence intervals on the CTSD estimates widened, providing accurate coverage for all but the coarsest sampling regimes (Fig. 5). We also found SLD and model-smoothed SLD estimates to become increasingly negatively biased as the amount of random data loss increased, whereas CTSD was, again, consistently accurate across the data loss regimes we examined (Fig. 4b).

Similarly, when the sampling frequency was fixed, SLD estimates varied substantially as the underlying movement differed, with, again, only a narrow window where the different sources of bias cancelled out to provided an accurate estimate. Model-smoothed SLD was generally more stable than conventional SLD, but did still suffer from scale-sensitivity, particularly for highly tortuous movement. In contrast, CTSD provided consistently accurate estimates, and was not biased by variation in tortuosity (Fig. 4c).

SLD estimates varied substantially as the underlying movement differed, with, again, only a narrow window where the different sources of bias cancelled out to provided an accurate estimate (Fig. 4c). In contrast, CTSD provided consistently accurate estimates, and was not biased by variation in tortuosity. Finally, as the amount of measurement error increased, the bias in SLD estimates, both conventional and model-smoothed, increased exponentially, whereas CTSD was not biased by measurement error (Fig. 4d).

**Fig. 6:**
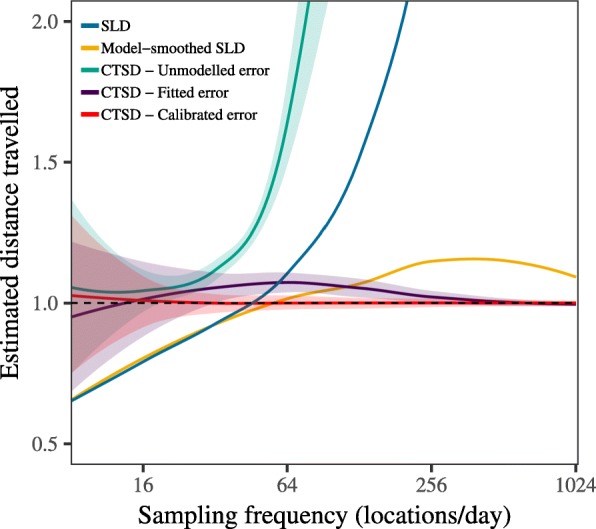
The results of simulations quantifying distance traveled via straight line displacement (SLD), and the continuous-time (CTSD) estimates from three different model fitting approaches; i) fitting the movement model without an error model; ii) fitting the movement and error models simultaneously; and iii) fitting the movement and error models separately via error calibration. The solid lines depict the mean accuracy, and the shaded areas the 95% CIs (SLD estimates, both model-smoothed and conventional, do not come with CIs). The dashed line at *y* = 1 depicts the true value to which the estimates should converge and the *x*-axis is log scaled

Importantly, while we found that CTSD, with a correctly specified error model, provided accurate estimates with reliable confidence intervals, CTSD with an incorrect error model resulted in inaccurate estimates (Fig. 6). For instance, when the movement model was fit without error, speed and distance estimates were even more biased that SLD estimates. Simultaneously fitting the movement and error models also resulted in biased estimates, though the extent of the bias was not as extreme as the scale-sensitive bias of conventional SLD estimation.

### Empirical results

**Fig. 7:**
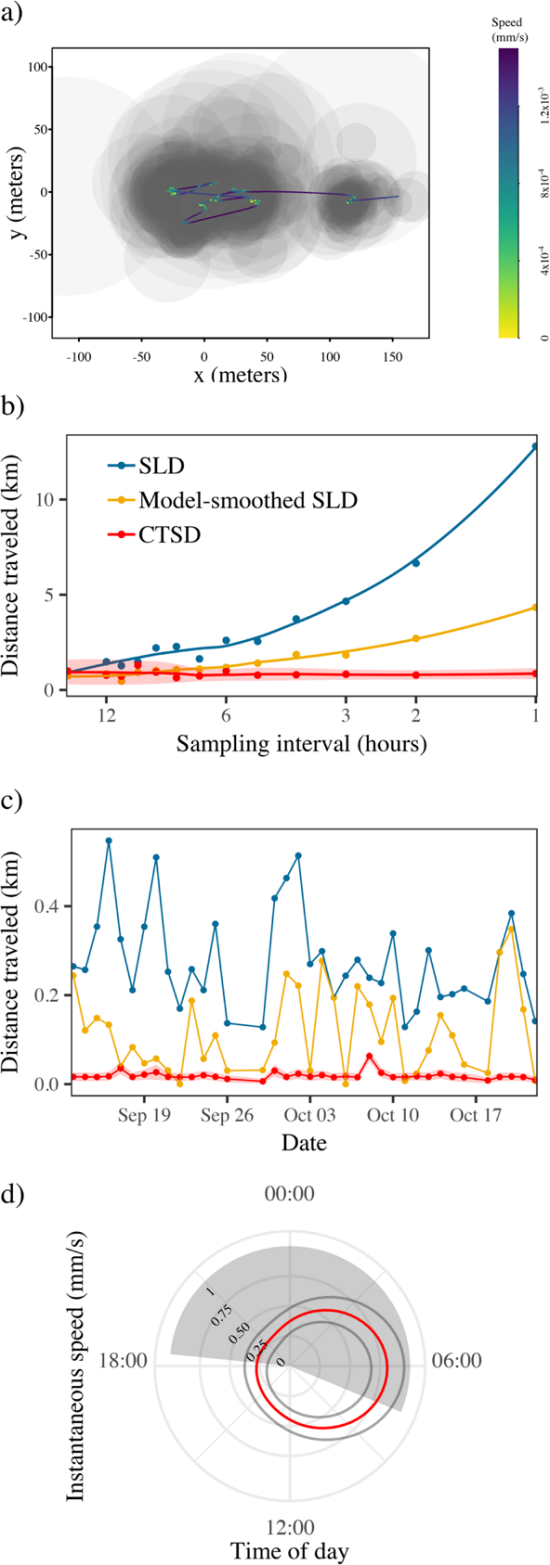
Figure depicting: **a** GPS data for a wood turtle (*Glyptemys insculpta*) tracked in Virginia, USA; (**b**) the total distance traveled estimated via conventional straight line displacement (SLD), model-smoothed SLD, and continuous-time speed and distance estimation (CTSD) approach using progressively thinned data; (**c**) the daily distance traveled again using conventional SLD, model-smoothed SLD, and CTSD; and (**d**) CTSD instantaneous speed estimates, ±95% CIs, averaged over a 24hr cycle. The gray circles in panel (**a**) depict the 50% error circles for GPS location estimates, the trajectory the most likely path between those locations, colored by the instantaneous speed estimates, while the gray shading in panel (**d**) depicts night time. Note how the measurement error is larger than the scale of the turtle’s movement (panel **a**) and, as a result, SLD estimates become dominated by error driven bias as the sampling frequency is increased (panel **b**), and vary substantially from day to day (panel **c**). Model-smoothing provided a reasonable, but insufficient correction to the error induced bias. In contrast, by accounting for the error structure of the telemetry data, the CTSD estimates are consistent across sampling frequencies, and suggest relatively consistent movement behavior throughout the study period. Panel (**d**) depicts how the turtle’s tends to move more in the early morning, with minimal movement throughout the rest of the day

Consistent with our simulated findings, SLD estimates of total distance traveled varied substantially with sampling frequency, whereas CTSD provided relatively consistent estimates except at very coarse sampling frequencies, but with appropriately wide confidence intervals. For instance, SLD estimation for the wood turtle’s tracking data at the full, 1 hr resolution, suggested this animal traveled 12.8 km over the 42 day sampling period, whereas CTSD estimated the distance traveled as 0.86 km (95% CIs: 0.57 – 1.15 km). Coarsening these data resulted in drastic changes to both of the SLD estimates (Fig. 7b), whereas CTSD point estimates and 95% CIs were all consistent. Interestingly, both of the scale-sensitive SLD estimates of daily movement distances varied substantially from day to day, whereas CTSD suggested relatively consistent behavior across the study period (Fig. 7c). The instantaneous speed estimates, averaged over each 24 h cycle, showed how the animal tended to move more in the early morning, with reduced movement throughout the rest of the day (Fig. 7d). SLD estimation does not readily allow for estimating instantaneous speeds from data that are coarse and irregular, precluding any formal comparison.

**Fig. 8:**
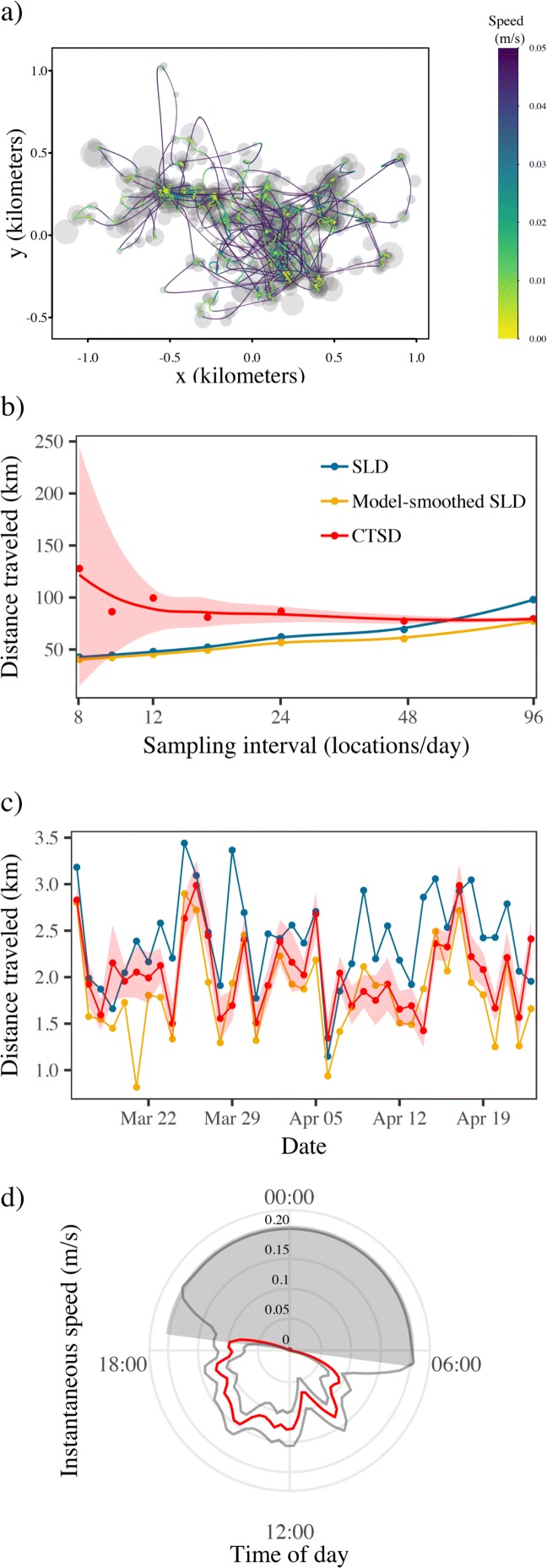
Figure depicting: **a** GPS data for a white nosed coati (*Nasua narica*) tracked on Barro Colorado Island, Panama; (**b**) the total distance traveled estimated via conventional straight line displacement (SLD), model-smoothed SLD, and continuous-time speed and distance estimation (CTSD) approach using progressively thinned data; (**c**) the daily distance traveled again using conventional SLD, model-smoothed SLD, and CTSD; and (**d**) CTSD instantaneous speed estimates, ±95% CIs, averaged over a 24hr cycle. The gray circles in panel (**a**) depict the 50% error circles for GPS location estimates, the trajectory the most likely path (MLP) between those locations, colored by the instantaneous speed estimates, while the gray shading in panel (**d**) depicts nighttime. Note how the animal’s trajectory does not necessarily move through the center of each location, as measurement error is accounted for when estimating the MLP. In panel (**d**) one can see how the coati tends to only move during daylight hours, and becomes stationary at night. However, note the appropriately wide CIs during the night time as the GPS unit was programmed to turn off after sundown

SLD estimation for the coati at the full, 15-min resolution suggested this animal traveled 97.9 km over the 41 day sampling period, whereas CTSD estimated the distance traveled as 79.5 km (95% CIs: 77.2 – 81.8 km). Again, iteratively coarsening these data resulted in more than a two-fold decrease in the SLD estimate (Fig. 8b), whereas CTSD point estimates and 95% CIs were all consistent, albeit with some positive bias and wide confidence intervals at the coarsest sampling frequencies. Similarly, there were significant differences in the daily distance traveled estimates between the two methods, where on only ca. 50% of the days were the SLD estimates within the 95% CIs of the CTSD estimates (Fig. 8c). The instantaneous speed estimates, averaged over each 24 h cycle, showed how the coati tended to move only during daylight hours, with a number of peak periods of activity, and little to no movement at night (Fig. 8d). This animal’s GPS collar was programmed to turn off at night, however. In this respect, note how the night time instantaneous speed estimates are accompanied by substantially wider confidence intervals than the daytime estimates, which is related to the large time-gap in the location data.

## Discussion

Speed and distance traveled are among the metrics most routinely estimated from GPS tracking data. Problematically however, the commonly used approach of estimating these using straight-line displacements is severely scale-sensitive, with biases arising from multiple sources [14–17, 22, 25, 58]. Even more problematic is the fact that each of these sources of bias operates in a different direction, and can be of variable magnitude. As the combination of sampling irregularities, inter-individual variation in movement, and measurement error are nearly ubiquitous aspects of animal tracking data, accurate speed/distance estimation requires statistical methods that can handle these complications, without being subject to artifactual differences due purely to estimator bias, or without having to know the magnitudes of these biases a priori to target the sampling rate accordingly. To date, corrections to these issues have included suggestions to increase the sampling frequency [16], *ad hoc* quantification of correction factors [17], and model-smoothing [26, 27]. These are unreliable solutions as they do not account for all sources of bias and also fail to provide a means of quantifying uncertainty in the estimates. While Johnson et al. [18] laid out a general approach to estimating trajectory-derived metrics, such as speed and distance traveled, by sampling from the posterior distribution of conditional trajectories, they did not implement this in readily accessible tools. The differences between our approach here and a hypothetical application of [18] are that we rely on a parametric bootstrap rather than treating the likelihood function as a Bayesian prior and we also take careful note from the recent results of [28] to not simultaneously fit movement and error parameters. In our view, it is unfortunate that the methods introduced by [18] have not been more widely adopted in movement ecology to date, while scale-sensitive SLD (whether model-smoothed or conventional) is still the estimator of choice for the majority of ecologists and practitioners.

As a solution to the outlined problems, we have developed CTSD as a new scale-insensitive method for estimating speed and distance traveled from animal tracking data that builds upon the existing continuous-time movement modeling framework [19, 30]. Using a combination of simulated and empirical data, we have demonstrated how CTSD provides accurate, scale-insensitive estimates with reliable confidence intervals, provided *Δt* is small enough to estimate *τ*_*v*_ (i.e., *Δt*<3*τ*_*v*_), and telemetry error is properly calibrated. The net results are speed and distance traveled estimates that can validly be compared across studies, sites, species, and times. For example, because the ∼15m median measurement error of the wood turtle’s tracking data was larger than the scale of the turtle’s movement over the 1 h sampling intervals (<1m), we found that the SLD estimates were dominated by error-driven bias. Consequently, the estimates varied more than 12-fold across the thinned sampling intervals, and when estimating the daily movement distances for this individual, the scale-sensitivity of the SLD resulted in estimates that varied substantially from one day to the next. The CTSD estimates in contrast, which accounted for the error structure of the telemetry data, suggested relatively consistent movement behavior throughout the study period. Had an analysis been based off of the SLD estimates, one would have erroneously concluded that this turtle covered large distances at highly variable rates, as opposed to the slow and steady movement it actually exhibited.

In the CTSD formalism, whole-path estimates, such as mean speed and distance traveled, are constructed from instantaneous speed estimates, which are also interesting in their own right. Instantaneous speeds averaged over cycles (e.g., 24hr, monthly, or seasonal cycles), such as those depicted in Figures 7d and Fig. 8d, can serve as the basis of visual diagnostic tools for identifying multiple behavioral states. When different behaviors are associated with clear differences in speed/velocity (e.g., active versus inactive, range-residency versus migration), instantaneous speed estimates can be used as the basis for formally estimating an individual’s behavioral state [10, 59]. For example, Fig. 7d shows how the turtle’s rate of movement changes throughout the day, with consistently more activity in the early morning, versus minimal movement throughout the rest of the day. Patterns in instantaneous speed over time can also allow researchers to identify the times and/or places where changes in movement and behavior occur [10].

While CTSD is, by itself, very general, it relies on a fitted movement model that adequately captures the underlying movement behavior in the data. In our experience, the current family of continuous-time models covers a very broad array of cases [19, 30, 38], that are useful for a wide range of species [40]. However, in cases where no appropriate model exists, then CTSD estimates may not be representative of the true speed/distance (for further details on how this may affect estimates see Additional file 5). The statistical efficiency of our method follows straightforwardly from related methods in time-series Kriging [60]. For a Gaussian stochastic process with a mean and autocorrelation function that are correctly specified by the movement model, the velocity estimates are minimum variance and unbiased (MVU; [61]). For non-Gaussian processes with correctly specified movement model, the velocity estimates are best linear unbiased estimates (BLUE; [61]). For asymptotic consistency, the movement model does not have to be correctly specified and only ‘compatibility’ (i.e., matching continuity) is required, but the variance of the errors does need to be correctly estimated [62] (see also Fig. 6). In other words, because speed and distance traveled are estimated as latent variables of the velocity parameter, asymptotic consistency requires a correlated velocity movement model where only the initial curvature of the model autocorrelation function needs to match that of the true autocorrelation function. The BLUE and asymptotic consistency properties of our method stand in contrast to the Gaussian mean-speed parameter estimates of [19], and [20], which are only accurate when the process is truly Gaussian. Moreover, the library of continuous-time movement models on which our method can be based is expanding rapidly [29, 32, 59, 63–65], including multi-state continuous-velocity models [66], so model misspecification should become less problematic going forward.

A further caveat to CTSD, and, indeed, any accurate method, is that it can not necessarily be applied to any dataset. If the data are too coarsely sampled, relative to the animal’s movement, to be able to fit a correlated velocity model [20], it will not be possible to estimate speed. This illustrates a fundamental aspect of studying movement through the use of tracking data, that when the sampling is too coarse to contain any signature of the animal’s velocity, this kind of analysis becomes inappropriate. For coarsely sampled data, although it is still mathematically possible to calculate the straight line displacement between any two locations, without a signature of *τ*_*v*_ these estimates are, ultimately, meaningless as measures of speed or distance traveled. In other words, just because an estimate can be produced when *Δt*>3*τ*_*v*_ does not mean said estimate is meaningful, as we demonstrate in Additional file 1. In this respect, the model selection step of our approach allows researchers to identify whether or not their data are of sufficient resolution to estimate these metrics in a statistically rigorous way. A corollary of this is that, if estimating speed/distance traveled is a primary goal of a study, we suggest researchers tailor their sampling design to ensure data of sufficient resolution to detect *τ*_*v*_. As a general rule of thumb, we suggest that the sampling interval should be less than or equal to *τ*_*v*_. On the other hand, because the effective sample size for velocity estimation, *N*_velocity_, corresponds to the equivalent number of statistically independent velocity observations, choosing a sampling interval much smaller than *τ*_*v*_ will produce marginal benefit. While *τ*_*v*_ is likely to differ between individuals, species, populations, seasons, etc., it tends to be on the order of minutes to hours for many range-resident species [19, 30, 50, 67]. In practice, sampling resolutions tend to be fine enough to estimate *τ*_*v*_ for the majority of GPS data for range-resident birds and mammals [40]. Although the empirical examples included in this work involved GPS data from terrestrial species, CTSD can can be applied to any form of tracking data (terrestrial, marine, avian, GPS, ARGOS, VHF, etc...) sampled at a finely enough to resolve *τ*_*v*_. Related to this, there will be some positive bias in the CTSD estimates when *τ*_*v*_ can not be accurately estimated, which happens when 3*τ*_*v*_>*Δt*>*τ*_*v*_. This is the result of small sample size bias, and happens because at coarse sampling frequencies, the ability to estimate *τ*_*v*_ is reduced and both the point estimate, and lower confidence interval on this parameter approach 0. CTSD uses the sampling distribution of $\hat {\tau }_{v}$ when parameterizing the simulations, so as more of this sampling distribution’s density becomes concentrated near zero, the simulated trajectories become more tortuous, and the estimated speed and/or distance traveled becomes increasingly large.

Our approach also requires being able to adequately account for measurement error in the data (i.e., by collecting calibration data, or by using pre-calibrated tracking devices). Without properly accounting for error, even CTSD with a perfectly specified movement model can result in arbitrarily biased speed/distance estimates. In this respect, while there is no substitute for true calibration data, there are viable alternatives if such data are not available. With GPS data, for instance, a default RMS UERE of 10-15m is often very reasonable — for example the wood turtle’s calibration estimated an RMS UERE of 10.6 meters. Furthermore, ‘opportunistic’ calibration data, like dead or sleeping animals can also be used in place of separately collected calibration data. Although these are viable alternatives, we do recommend that the collection of error calibration data becomes a standard component of future animal tracking studies.

## Conclusion

In conclusion, the methods developed in this study allow for the scale-insensitive estimation of mean speed, instantaneous speeds, and distance traveled from animal tracking data, that can correct for the often massive biases introduced by the sampling frequency [14, 15, 22], the tortuosity of the animal’s movement [16], and the amount of measurement error [25, 58], provided *Δt*>3*τ*_*v*_ and measurement error can be properly accounted for. In addition to being statistically rigorous, CTSD also benefits from being computationally efficient, a property that is well suited to the growing volume of data used in these analyses [13]. All the methods developed in this study are now freely available in the R package ctmm (version 0.5.7; [19]) via the speed() and speeds() functions, or through the point-and-click web based graphical user interface at ctmm.shinyapps.io/ctmmweb/ (version 0.2.5; [48]).

## Supplementary information

**Additional file 1** Proofs of SLD biases. Mathematical proof of the two SLD biases—overestimation at small sampling interval *Δt* and underestimation at large sampling interval, as well as a additional simulation based results demonstrating SLD’s inability to return an accurate estimate when the sampling interval, *Δt*, is longer the the velocity autocorrelation timescale *τ*_*v*_.

**Additional file 2** Workflow for estimating speed and distance traveled using CTSD in ctmm.

**Additional file 3** Technical details. Details on estimating the mean speed and root mean square (RMS) speed from either a time-averaged stationary Gaussian stochastic process or from instantaneous Kriged velocity estimates, and how we translate point estimates and standard errors into non-standard confidence intervals.

**Additional file 4** R script for reproducing the simulations.

**Additional file 5** CTSD and model misspecification. Evaluations of the performance of CTSD with misspecified models for scenarios that are likely to occur in real data.

## Data Availability

The white-nosed coati data used in this manuscript are available from the Movebank online repository (DOI: 10.5441/001/1.41076dq1), the wood turtle and brown pelican data are included in the ctmm package, and the source code for the ctmm package is available on CRAN.

## Abbreviations

BLUE: best linear unbiased estimates
ctmm: continuous-time movement modelling
CTSD: Continuous-Time Speed and Distance
DOP: Dilution of Precision
GPS: Global Positioning System
HDOP: Horizontal Dilution of Precision
IOU: Integrated Ornstein-Uhlenbeck
MVU: Minimum Variance and Unbiased
OU: Ornstein-Uhlenbeck
OUF: Ornstein-Uhlenbeck Foraging
RMS: Root Mean Square
SLD: Straight Line Displacement
UERE: User Equivalent Range Error

## Footnotes

1 More computationally efficient numerical integrators exist, but they require evenly sampled data.
