# Supplementary material for: Scale-insensitive estimation of speed and distance traveled from animal tracking data
Source: Mov Ecol. 2019 Nov 15;7:35. doi: 10.1186/s40462-019-0177-1 (PMC6858693; doi:10.1186/s40462-019-0177-1)
Supplement: Supplementary file 1 — Additional file 1 Proofs of SLD biases. Mathematical proof of the two SLD biases—overestimation at small sampling interval Δt and underestimation at large sampling interval, as well as a additional simulation based results demonstrating SLD’s inability to return an accurate estimate when the sampling interval, Δt, is longer the the velocity autocorrelation timescale τv. [file 40462_2019_177_MOESM1_ESM.pdf]

Additional file S1 — Proofs of SLD biases

In this appendix we provide we provide mathematical proof of the two SLD biases—overestimation at small sampling interval Δt and underestimation at large sampling interval, as well as a additional simulation based results demonstrating SLD's inability to return an accurate estimate when the sampling interval, Δt, is longer the the velocity autocorrelation timescale τ_v.

Mathematical Proofs

For this proof we we assume a fairly general model of location observations with additive error

$$\mathbf{x}_{\text{obs}}(t) = \underbrace{\boldsymbol{\mu}(t) + \boldsymbol{\xi}(t)}_{\text{true } \mathbf{x}(t)} + \underbrace{\boldsymbol{\epsilon}(t)}_{\text{error}}, \tag{1}$$

where $\boldsymbol{\mu}(t) = \langle \mathbf{x}(t) \rangle$ corresponds the deterministic motion, $\boldsymbol{\xi}(t) = \mathbf{x}(t) - \boldsymbol{\mu}(t)$ corresponds to the stochastic motion, and $\boldsymbol{\epsilon}(t)$ denotes the telemetry error. For simplicity, we further assume that $\boldsymbol{\epsilon}(t)$ is a mean-zero IID process and $\boldsymbol{\xi}(t)$ is a stationary process, so that the true location $\mathbf{x}(t)$ is a trend-stationary process.

The SLD speed estimate at a midpoint time (where the estimation error is smallest) is given by

$$\hat{v}(t) = \frac{|\mathbf{x}_{\text{obs}}(t{+}\Delta t/2) - \mathbf{x}_{\text{obs}}(t{-}\Delta t/2)|}{\Delta t}, \tag{2}$$

but instead we focus on the pathological biases in the SLD square-velocity estimate

$$\hat{v}(t)^2 = \frac{|\mathbf{x}_{\text{obs}}(t{+}\Delta t/2) - \mathbf{x}_{\text{obs}}(t{-}\Delta t/2)|^2}{\Delta t^2}, \tag{3}$$

as for this estimator we can perform an exact analysis without making any distributional assumptions on the movement or telemetry-error processes. Importantly, if the mean square velocity estimates can be shown to diverge for small Δt and vanish for large Δt, then the mean speed estimates must do the same.

Taking the expectation value $\langle \cdots \rangle$, we have after some simplification the mean square velocity estimate

$$\langle \hat{v}(t)^2 \rangle = \frac{|\boldsymbol{\mu}(t{+}\Delta t/2) - \boldsymbol{\mu}(t{-}\Delta t/2)|^2}{\Delta t^2} + 2\,\mathrm{tr}\left[\frac{\boldsymbol{\sigma}(0) - \boldsymbol{\sigma}(\Delta t)}{\Delta t^2}\right] + 2\,\mathrm{tr}\left[\frac{\mathrm{COV}[\boldsymbol{\epsilon}(t)]}{\Delta t^2}\right], \tag{4}$$

where $\boldsymbol{\sigma}(\tau) = \mathrm{COV}[\mathbf{x}(t{+}\tau), \mathbf{x}(t)]$ denotes the autocorrelation function of the movement process, and tr[M] refers to the matrix trace. We note that when Δt becomes large, then this expectation value limits to zero, which implies underestimation at large values of Δt. One the other hand, when Δt becomes small, then the third, telemetry-error term clearly diverges, which implies overestimation

at small values of Δt. Next we perform a more detailed analysis to determine the characteristic scales at which these biases emerge. In the first, deterministic term of (4) we taylor expand $\boldsymbol{\mu}(t\pm\Delta t/2)$ around the midpoint time t to obtain

$$\langle \hat{v}(t)^2 \rangle = \dot{\boldsymbol{\mu}}(t)^2 + 2\,\mathrm{tr}\left[\frac{\boldsymbol{\sigma}(0) - \boldsymbol{\sigma}(\Delta t)}{\Delta t^2}\right] + 2\,\mathrm{tr}\left[\frac{\mathrm{COV}[\boldsymbol{\epsilon}(t)]}{\Delta t^2}\right] + \frac{\dddot{\boldsymbol{\mu}}(t)^2}{144}\Delta t^4 + \mathcal{O}(\Delta t^8), \tag{5}$$

where $\dot{x}(t) = \frac{d}{dt}x(t)$ denotes differentiation with respect to time. Next, to simplify the second, stochastic term of (5), we use the kinematic Taylor expansion of the autocorrelation function at lag zero

$$\boldsymbol{\sigma}(\Delta t) = \mathrm{COV}[\mathbf{x}(t)] - \frac{1}{2}\mathrm{COV}[\dot{\mathbf{x}}(t)]\,\Delta t^2 + \frac{1}{24}\mathrm{COV}[\ddot{\mathbf{x}}(t)]\,\Delta t^4 + \mathcal{O}(\Delta t^6)\,, \tag{6}$$

which can be obtained from the regular Taylor expansion after applying the lag derivatives to the stochastic processes inside the expectation value. This expansion represents the autocorrelation at lag Δt in terms of the covariance in location, velocity, acceleration, etc.. Inserting this expansion in to (5), we finally have the mean square velocity estimate

$$\langle \hat{v}(t)^2 \rangle = \underbrace{\dot{\boldsymbol{\mu}}(t)^2 + \mathrm{tr}[\mathrm{COV}[\dot{\mathbf{x}}(t)]]}_{\text{true } \langle v(t)^2 \rangle} + \underbrace{2\,\mathrm{tr}\left[\frac{\mathrm{COV}[\boldsymbol{\epsilon}(t)]}{\Delta t^2}\right]}_{\frac{1}{\Delta t^2}\text{ observation error}} \underbrace{-\,\mathrm{tr}\left[\frac{\mathrm{COV}[\ddot{\mathbf{x}}(t)]}{12}\right]\Delta t^2}_{\Delta t^2 \text{ tortuosity error}} + \mathcal{O}(\Delta t^4). \tag{7}$$

Therefore, when the sampling interval is short, then SLD estimators acquire positive bias from measurement error, whereas when the sampling interval is long, then SLD estimators acquire negative bias from acceleration-induced tortuosity. For SLD estimators to be accurate, they must then satisfy the 'Goldilocks's' criterion

$$\underbrace{\sqrt{\frac{\mathrm{tr}\,\mathrm{COV}[\boldsymbol{\epsilon}(t)]}{\langle v(t)^2 \rangle}}}_{\tau_{\mathrm{err}}} \ll \Delta t \ll \underbrace{\sqrt{\frac{\langle v(t)^2 \rangle}{\mathrm{tr}\,\mathrm{COV}[\ddot{\mathbf{x}}(t)]}}}_{\tau_{\mathrm{tor}}}, \tag{8}$$

which is not generally possible, as it requires the product of telemetry error and tortuosity to be sufficiently small

$$\sqrt{\mathrm{tr}\,\mathrm{COV}[\boldsymbol{\epsilon}(t)]\,\mathrm{tr}\,\mathrm{COV}[\ddot{\mathbf{x}}(t)]} \ll \langle v(t)^2 \rangle\,, \tag{9}$$

in addition to the sampling interval Δt being tuned between the two scales τ_{err} and τ_{tor}.

Simulations

Here, we use a set of simulations to further illustrate that requiring sampling that is fine-scale enough to resolve the velocity autocorrelation time-scale, τ_v, is not a limitation of CTSD, specifically, but a universal, albeit under-recognized, limitation of the data. When animal movement is

sampled so coarsely that no signature of the underlying movement path remains in the data, no method will provide a reliable estimate of speed and/or distance travelled. For these simulations, we sequentially manipulated both τ_v and the sampling interval, Δt, while holding all else equal, using the same simulation protocols described in Appendices S1 and S3. Furthermore, we did not add any telemetry error to the data, but instead assumed perfect measurement accuracy. When then performed SLD estimation on these data and evaluated the capacity to obtain an unbiased estimate when $\Delta t > \tau_v$ or $\Delta t < \tau_v$.

The results of these simulations are presented in figure 1. We see that although SLD on the true locations can result in accurate estimate when $\Delta t < \tau_v$, when $\Delta t > \tau_v$ SLD only ever returns a biased estimate, even with perfectly sample location data. In other words, when $\Delta t > \tau_v$, although it is still mathematically possible to calculate the straight line distance between any two locations, without a signature of the underlying movement path in the data these estimates are, ultimately, meaningless as measures of speed or distance travelled. In this respect, the model selection step of CTSD allows researchers to identify whether or not their data are of a sufficient resolution to estimate these metrics in a way that can return estimates with meaningful information.

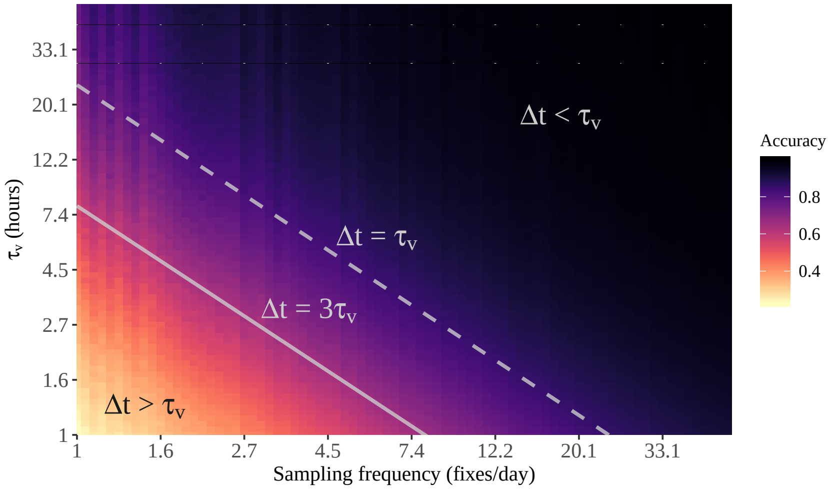

Figure 1: The results of simulations demonstrating the inability to obtain an accurate estimate via straight line distance (SLD) when the sampling interval, Δt, is longer the the velocity autocorrelation timescale, τ_v.
