# Supplementary material for: Scale-insensitive estimation of speed and distance traveled from animal tracking data
Source: Mov Ecol. 2019 Nov 15;7:35. doi: 10.1186/s40462-019-0177-1 (PMC6858693; doi:10.1186/s40462-019-0177-1)
Supplement: Supplementary file 2 — Additional file 2 Workflow for estimating speed and distance traveled using CTSD in ctmm. [file 40462_2019_177_MOESM2_ESM.pdf]

Additional file S2 — Workflow for estimating speed and distance travelled using CTSD in `ctmm`

In this appendix we provide three worked examples of how to estimate mean speed over the study period, instantaneous speed at sampled times, and daily distance travelled, using the continuous-time latent speed and distance estimation (CTSD) method detailed in the main text. These are carried out on GPS data from a white-nosed coati (*Nasua narica*) tracked on Barro Colorado Island, Panama [1], and a wood turtle (*Glyptemys insculpta*) tracked in Virginia, USA, and ARGOS data from a brown pelican *Pelecanus occidentalis* tracked on the eastern coast of the United States. These three datasets are openly available within the `ctmm` package.

White-nosed coati example:
Estimating speed/distance with a pre-calibrated device

The coati tracking data used in the main text to demonstrate the functionality of CTSD were collected using an e-obs collar. These devices have pre-calibrated dilution of precision (DOP) values, and the data come with 'eobs horizontal accuracy estimate' (in meters) as opposed to HDOP and VDOP columns respectively. As such, there is no need to collect calibration data to calibrate the device's error prior to analysis.

```
#Load the package
library(ctmm)

#Load in the coati tracking dataset
data("coati")

#Extract the data from the desired individual
#and return some summary statistics
DATA <- coati[[1]]

summary(DATA)

$identity
[1] "Aleja"

$timezone
[1] "UTC"

$projection
[1] "+proj=tpeqd +lon_1=-79.8508054092888 +lat_1=9.1520090169231
     +lon_2=-79.8466077969879 +lat_2=9.15236182525117 +datum=WGS84"
```

```
$`sampling interval (minutes)'
[1] 15.18333

$`sampling period (months)'
[1] 1.351685

$`longitude range'
[1] -79.85899 -79.83657

$`latitude range'
[1] 9.147379 9.164963

#Plot the tracking data showing the 50% error circles
plot(DATA,
     error = 2,
     level.UD = 0.50)
```

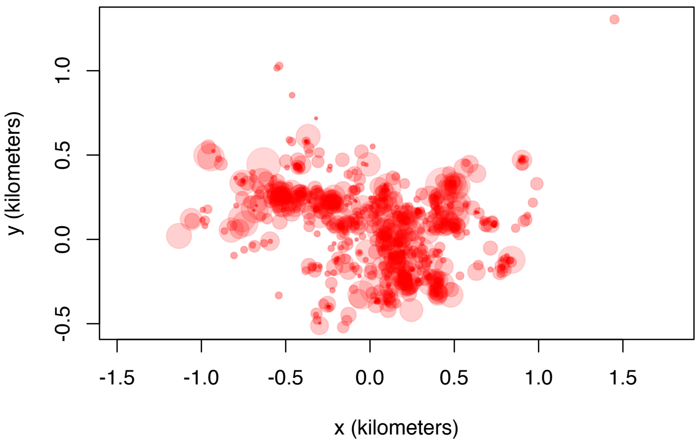

Figure 1: A scatterplot of the GPS positional observations for a white nosed coati (*Nasua narica*) tracked on Barro Colorado Island, Panama. Data were collected using e-obs collars collecting locations at a 15 minute sampling interval, over 41 days.

Before fitting any movement models to these data, the first step is to ensure that there are no outliers that could bias parameter estimation. This step is facilitated by the `outlie()` function in the `ctmm` package (Fleming et al. *in prep.*). This function calculates distances from the median longitude and latitude, and maximum speeds over each time step. It returns a `data.frame` of these estimates, as well as a plot with intervals of high speed highlighted with blue segments, and

distant locations highlighted with red points. Both estimates account for telemetry error and the speed estimates account for timestamp truncation and assign each time step's speed to the most likely offending time (based on the speed estimate of adjacent locations). We note that the speed estimates used here are tailored for outlier detection and have poor statistical efficiency.

```
#Check for any outliers
OUTLIERS <- outlie(DATA)
```

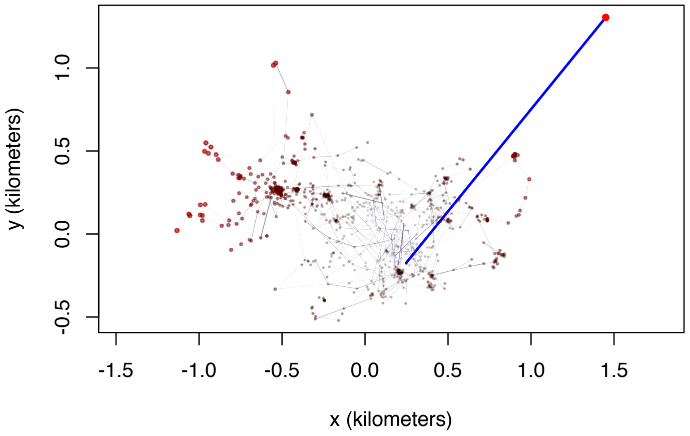

Figure 2: A scatterplot of the GPS positional observations returned by the `outlie()` function. Intervals of high speed are highlighted with blue segments, and distant locations are highlighted with red points

The location in the upper right-hand of the plot appears to be an outlier, but, to confirm this, it is useful to plot the speed and distance estimates.

```
plot(OUTLIERS)
```

This scatterplot confirms our suspicion that the location in question is an outlier, and we can proceed to remove it.

```
#Remove the outlier based on the estimated speed
DATA <- DATA[-(which(OUTLIERS[[1]] >= 0.6)),]
```

Finally, to confirm we have removed all of the outliers, we repeat the above steps on the filtered data.

```
#Re-check for any remaining outliers
par(mfrow=c(1,3))
```

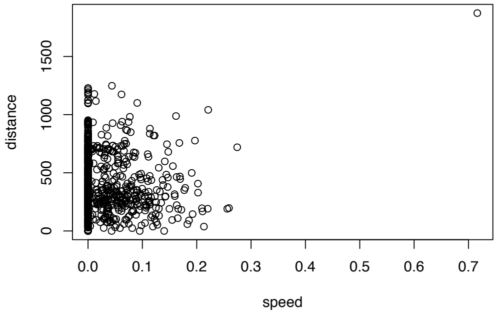

Figure 3: A scatterplot of the speed and distance estimates returned by the `outlie()` function. Note how the majority of the estimates are clustered together, but with one obvious outlier in the upper right-hand of the plot.

```
plot(DATA,
     error = 2,
     level.UD = 0.50)

OUTLIERS <- outlie(DATA)

plot(OUTLIERS)
```

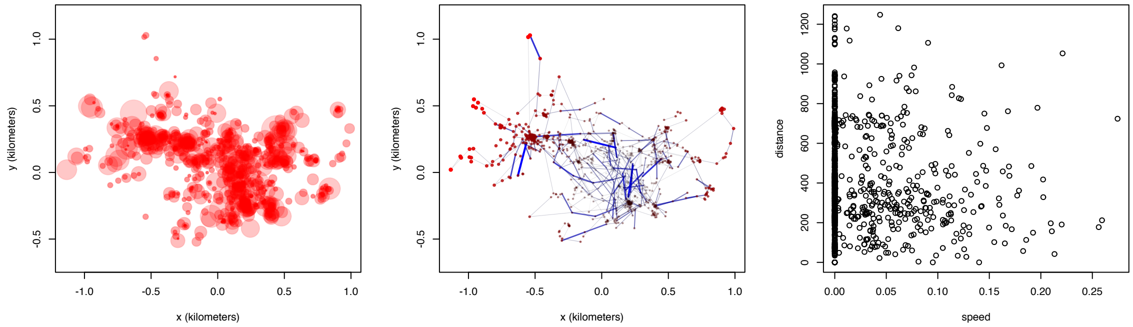

Figure 4: From left to right, scatterplots of the GPS positional observations, the GPS positional observations returned by the `outlie()` function, and the speed and distance estimates returned by the `outlie()` function. Note how there is not longer any indication of outliers in the data.

After removing any outliers from the data, the next step is to fit a series of continuous-time

movement models to the data, and the best fit model is selected based on the approximate small-sample-size corrected Akaike Information Criterion [for a complete description of the model selection step see: 2].

```
#Generate the variogram
vg <- variogram(DATA)

#Guesstimate the model to obtain initial parameter values
GUESS <- ctmm.guess(DATA,
                    variogram = vg,
                    interactive = FALSE)

#Turn error on
GUESS$error <- TRUE

#Fit and select models
FITS <- ctmm.select(DATA,
                    CTMM = GUESS)

#Return a summary of the selected model
summary(FITS)

$name
[1] "OUF isotropic error"

$DOF
     mean      area     speed
 41.00426  68.62914 537.66568

$CI
                               low        ML      high
area (square kilometers)  1.601182  2.059400  2.574401
τ[position] (hours)       8.121016 11.089522 15.143118
τ[velocity] (minutes)    18.627999 21.661519 25.189039
speed (kilometers/day)    5.373700  5.610866  5.847895
#Note: the speed value returned here is the RMS speed
#detailed in Appendix S1


#Plot the variogram and selected model to visualy inspect the fit
plot(vg,
     CTMM = FITS)
```

With filtered data, and an appropriate movement model in hand, the final step is to estimate the animal's instantaneous speeds, the mean speed over the study period, or the speed/distance travelled over a specific period of time.

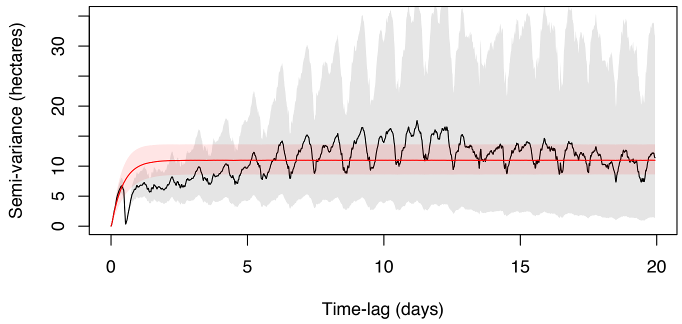

Figure 5: Variogram derived from the coati's location data. The black line and grey shading depict the semi-variance ± 95% CIs, whereas the red line and shading depict the fitted movement model ± 95% CIs of the model fit.

```
#Estimate mean speed over the duration of the study period
speed(DATA,
      FITS)

                                  low       ML     high
speed (kilometers/day) 4.305776 4.439711 4.57465


#Estimate the instantaneous speeds
SPEEDS <- speeds(DATA,
                 FITS)


head(SPEEDS)

          low          ML        high          t           timestamp
1 0.007752602 0.04316145 0.09353133 1268670712 2010-03-15 16:31:52
2 0.005661690 0.03153354 0.06833981 1268672450 2010-03-15 17:00:50
3 0.005020705 0.02795043 0.06056812 1268673356 2010-03-15 17:15:56
4 0.005774482 0.03209965 0.06953654 1268674253 2010-03-15 17:30:53
5 0.007557332 0.04179921 0.09044595 1268675130 2010-03-15 17:45:30
6 0.008125551 0.04512254 0.09772508 1268679695 2010-03-15 19:01:34

###############################################################
####   Estimating daily movement distance over a study period

#First identify how many days the individual was tracked for
```

```
DATA$day <-cut(DATA$timestamp,breaks="day")

days <- unique(DATA$day)



#An empty list to fill with the results
res <- list()

#Loop over the number of days
for(i in 1:length(days)){

  message("Estimating distance travelled on day ", i,": ", days[i])

  #Select data for the day in question
  DATA.SUBSET <- DATA[which(DATA$day == days[i]),]

  #Calculate the duration of the sampling period (in seconds)
  SAMP.TIME <- diff(c(DATA.SUBSET$t[1],
                      DATA.SUBSET$t[nrow(DATA.SUBSET)]))

  #Guesstimate the model for initial parameter values
  GUESS <- ctmm.guess(DATA,
                      variogram = variogram(DATA),
                      interactive = FALSE)

  #Turn error on
   GUESS$error <- TRUE

  #Fit the movement model to the day's data
   FITS <- ctmm.fit(DATA.SUBSET,
                    CTMM = GUESS)

  #Calculate speed in m/s
  ctmm_speed <- speed(object = DATA.SUBSET,
                     CTMM = FITS,
                     units = FALSE)


  #Multiply speed (in m/s) by the sample time (in s)
  #to get the estimated distance travelled (in m)
  ctmm_dist <- ctmm_speed*SAMP.TIME

  #Re-name the variable
  rownames(ctmm_dist) <- "distance (meters)"
```

```
  #And store the results in the list
  x <- c(i, #The day
         ctmm_dist[2], #The ML distance estimate
         ctmm_dist[1], #Min CI
         ctmm_dist[3]) #Max CI

  names(x) <- c("date", "dist.ML", "dist.Min", "dist.Max")

  res[[i]] <- x
}

#Finally bind results together as a data frame
res <- as.data.frame(do.call(rbind, res))
res$date <- as.Date(days)

head(res)

        date   dist.ML  dist.Min  dist.Max
1 2010-03-15  351.2639  255.0523  454.6734
2 2010-03-16 3109.6675 2853.1944 3371.4784
3 2010-03-17 2068.6985 1813.2098 2332.2601
4 2010-03-18 1837.9638 1686.7342 1992.3331
5 2010-03-19 2426.6460 2005.2749 2867.0971
6 2010-03-20 2168.0942 1889.9962 2455.3340
```

Wood turtle example: Estimating speed/distance with calibration data

For the wood turtle's data, locations were collected using a tracking device that did not have calibrated error. For these data, the first step is to therefore calibrate the DOP values. This is done by using calibration data, where the tracking device has been left in a fixed location for a period of time, to estimate the device's user equivalent range error (UERE). The device specific UERE is then used to translate the unit-less GPS dilution of precision (DOP) values into standard deviations of mean-zero error, where the horizontal error = UERE $\times$ HDOP [3]. Calibration data for this turtle's tracking tag were collected by leaving two devices in a fixed location, each for 1 day, sampling at 10-minute intervals. The `uere.fit()` function from the `ctmm` package is then used to both estimate the UERE, and the `uere()<-` function was used to assign that value to the tracking data.

```
#Load in the wood turtle calibration, and tracking data
data("turtle")

#Split the data into the calibration datasets
#And the desired individual's tracking data
```

```
CALIBRATION <- list(turtle[1:2])

DATA <- turtle[[4]]

#Plot the calibration data
plot(CALIBRATION,
     error = 2,
     level.UD = 0.5,
     ylim = c(-200, 200))
```

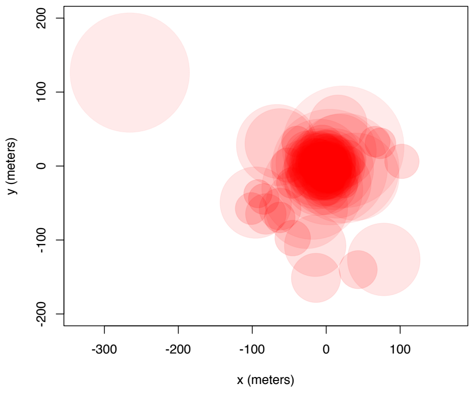

Figure 6: Scatterplot of the device's calibration data.

```
#Estimate the UERE
UERE <- uere.fit(CALIBRATION)

summary(UERE)

An object of class "UERE"
    horizontal
all   10.62878
Slot "DOF":
    horizontal
all        229

Slot "AICc":
horizontal
  3993.712

Slot "Zsq":
horizontal
```

```
  6.179499

#Assign the UERE to the tracking data
uere(DATA) <- UERE

#Plot the tracking data showing the 50% error circles
plot(DATA,
     error = 2,
     level.UD = 0.50)
```

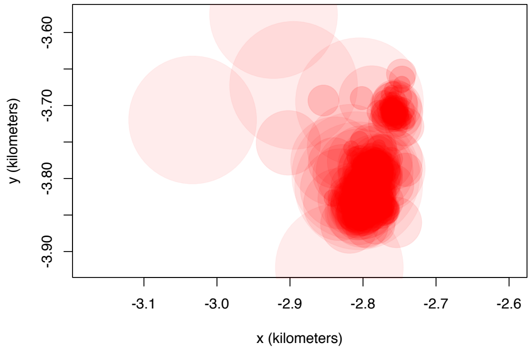

Figure 7: A scatterplot of the wood turtle's GPS positional observations.

After having calibrated the errors, the process of estimating speed and distance travelled using the CTSD approach is much the same as that detailed for the coati's tracking data above. First, any outliers are removed from the dataset, movement models are fit to the clean data, and the best fit model is used to generate the desired estimates.

```
#Identify any potential outliers and visualise the outputs
par(mfrow=c(1,2))
OUTLIERS <- outlie(DATA)

plot(OUTLIERS)

#Filter out the outliers
DATA <- DATA[-(which(OUTLIERS[[1]] >= 0.03)),]

#Generate the variogram
vg <- variogram(DATA)

#Guesstimate the model for initial parameter values
GUESS <- ctmm.guess(DATA,
```

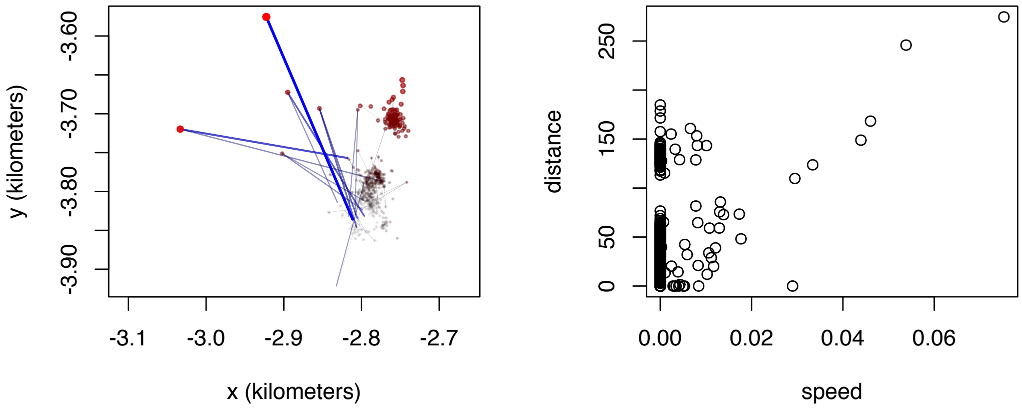

Figure 8: From left to right, the GPS positional observations returned by the `outlie()` function, and the speed and distance estimates returned by the `outlie()` function.

```
                              variogram = vg,
                              interactive = FALSE)

#Turn error on
GUESS$error <- TRUE

#Fit and select models
FITS <- ctmm.select(DATA,
                              CTMM = GUESS)

#Return a summary of the selected model
summary(FITS)

$name
[1] "OUF anisotropic error"

$DOF
      mean       area      speed
  1.752473   1.733970  13.040823

$CI
                                    low          ML        high
area (hectares)         0.2486431   2.610812   7.682437
τ[position] (days)      0.0000000  26.570201  66.986488
```

```
τ[velocity] (hours)  1.3185238  7.787562 45.995469
speed (meters/day)  18.9896098 26.009084 33.015861
#Note: the speed value returned here is the RMS speed
#detailed in Appendix S1

#Estimate mean speed over the duration of the study period
speed(DATA,
      FITS)

                         low       ML     high
speed (meters/day) 13.72984 19.26969 25.24772


#Estimate the instantaneous speeds in m/s
SPEEDS <- speeds(DATA,
                 FITS)


head(SPEEDS)

            low            ML          high     t
1 0.00001883583 0.0002334009 0.0005892840     0
2 0.00001879900 0.0002276023 0.0005724466  3604
3 0.00001860172 0.0002205374 0.0005527448  7200
4 0.00001837331 0.0002133778 0.0005329544 10799
5 0.00001814747 0.0002068077 0.0005149008 14402
6 0.00001779611 0.0002004074 0.0004979616 18001
```

Brown pelican (*Pelecanus occidentalis*) example: Estimating speed/distance with ARGOS data

The empirical tracking data used in the main text to demonstrate the functionality of CTSD were collected using GPS-based tags. While GPS data tend to have greater positional accuracy and finer resolutions than data collected using ARGOS based telemetry, CTSD can be applied to ARGOS data. Here, we walk through an application of the method on data for a brown pelican *Pelecanus occidentalis* collected using tags that collected both ARGOS and GPS data (Fig. 9). A total of 338 ARGOS-based locations, and 1295 GPS-based were sampled over a ∼5 month period in winter 2017. The ARGOS based locations had a mean location error of 414.5 meters (ranging from 132 m – 3.7 km), while the GPS based locations had a mean HDOP of 3.2 (ranging from 2 – 10). Modern ARGOS devices come pre-calibrated, so, for these data, no additional data collection and/or calibration was necessary. ARGOS data and the error ellipse information are automatically imported into `ctmm`. For the GPS data, however, no calibration data were available. As such, we set the RMS UERE to 10m.

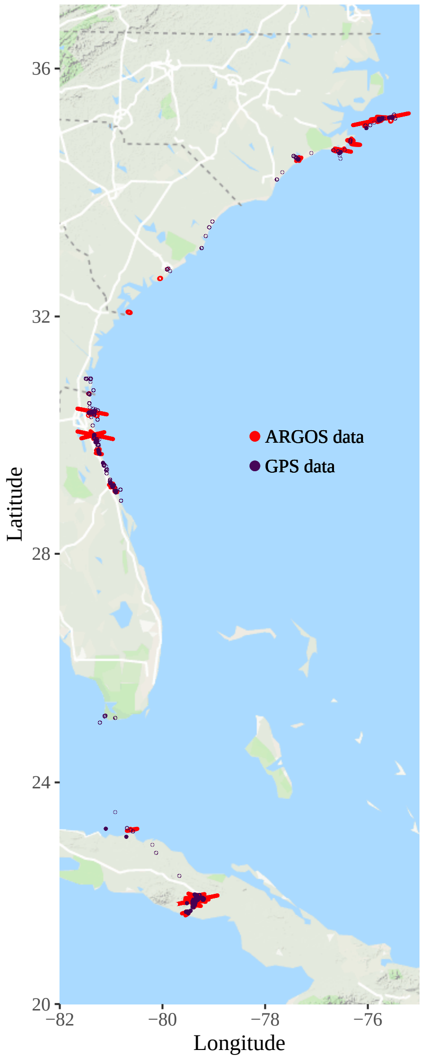

Figure 9: A scatterplot of the positional observations for a brown pelican *Pelecanus occidentalis* tracked on the eastern coast of the United States. Data were collected using a tag that recorded 338 ARGOS locations, and 1295 GPS locations over a ∼5 month period.

```
#Load in the brown pelican tracking data
data("pelican")

summary(pelican)

$identity
```

```
[1] "BRPE_03_2017_Adam"

$timezone
[1] "UTC"

$projection
[1] "+proj=tpeqd +lon_1=-79.3445351593173 +lat_1=21.9507561869621 +lon
   _2=-76.1854133925203 +lat_2=38.2184417075292 +datum=WGS84 +ellps=
   WGS84 +towgs84=0,0,0"

$`sampling interval (hours)`
[1] 1.185139

$`sampling period (months)`
[1] 5.169918

$`longitude range`
[1] -81.44378 -75.55172

$`latitude range`
[1] 21.63308 35.22516

#Check for any outliers
OUTLIERS <- outlie(pelican)
```

There are no locations that appear to be obvious outliers, but, to confirm this, it is useful to plot the speed and distance estimates.

```
plot(OUTLIERS)
```

This scatterplot confirms our suspicion that there are no obvious outliers in the data, and we can proceed with the analyses.

The next step is to fit a series of continuous-time movement models to the data, and the best fit model is selected based on the approximate small-sample-size corrected Akaike Information Criterion.

```
#begin by returning some summary statistics on the data

#Generate an initial guess for an IOU (non-range resident) model
GUESS <- ctmm.guess(argos_data,
                    interactive = FALSE,
                    CTMM = ctmm(tau = c(Inf, 2 %#% 'hours'),
                                range = FALSE))


#Turn error on
GUESS$error <- TRUE
```

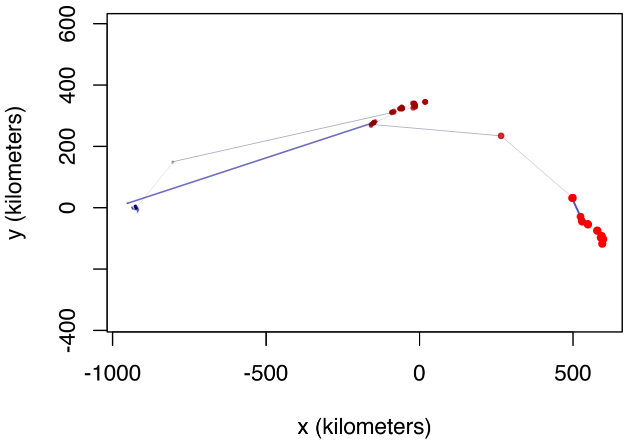

Figure 10: A scatterplot of the ARGOS positional observations returned by the `outlie()` function. Intervals of high speed are highlighted with blue segments, and distant locations are highlighted with red points

```
#Fit and select models
argos_FIT <- ctmm.select(pelican,
                         GUESS)

#Return a summary of the selected model
summary(argos_FIT)

$name
[1] "IOU anisotropic error"

$DOF
    mean      area    speed
  0.0000    0.0000 194.6332

$CI
                               low        est        high
τ[velocity] (hours)        1.85794   2.304798    2.859132
speed (kilometers/day) 150.82847 162.224325 173.609543
#Note: the speed value returned here is the RMS speed
#detailed in Appendix S1
```

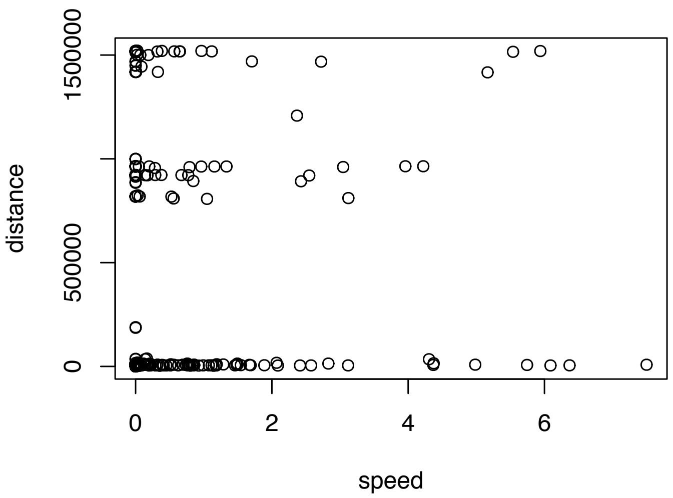

Figure 11: A scatterplot of the speed and distance estimates returned by the `outlie()` function. Note how the majority of the estimates are clustered together, with no obvious outliers.

```
#Plot the variogram and selected model to visualy inspect the fit
plot(variogram(argos_data,
               fast = FALSE,
               dt = (c(1,6,24) %#% "hr")),
     CTMM = argos_FIT)
```

With outlier free data, and an appropriate movement model in hand, the final step is to estimate the animal's instantaneous speeds, the mean speed over the study period, or the speed/distance travelled over a specific period of time.

```
#Estimate mean speed over the duration of the study period
speed(pelican,
      argos_FIT)

                             low      est     high
speed (kilometers/day) 87.58893 93.43971 99.38238


#Estimate the instantaneous speeds
argos_SPEEDS <- speeds(pelican,
                       argos_FIT)
```

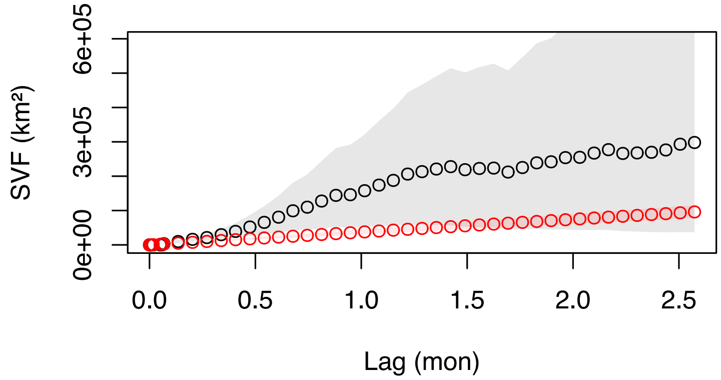

Figure 12: Variogram derived from the pelican's location data. The black line and grey shading depict the semi-variance ± 95% CIs, whereas the red line and shading depict the fitted movement model ± 95% CIs of the model fit. Note the lack of a clear asymptote in the empirical variogram.

```
head(argos_SPEEDS)

        low       est     high          t           timestamp
1 4.4141978 5.6301304 6.915797 1513289368 2017-12-14 22:09:28
2 2.7031713 3.6199390 4.599460 1513292364 2017-12-14 22:59:24
3 0.1105584 0.6354424 1.386813 1513295439 2017-12-14 23:50:39
4 0.2301657 0.9992814 2.040662 1513298604 2017-12-15 00:43:24
5 0.1711297 1.0425512 2.304380 1513305604 2017-12-15 02:40:04
6 0.2432888 1.4751163 3.257147 1513475824 2017-12-17 01:57:04
```

In comparison the SLD estimated speed for this individual can be estimated as:

```
#The SLD Speed (in km/day)
#First get the duration of the sampling period (in days)
DURATION <- (tail(pelican$t,1) - head(pelican$t,1))/86400

#The SLD speed (distance/time)
(tot.dist(pelican)/1000)/DURATION

27.32517
```

For these coarsely sampled ARGOS data, CTSD estimation suggested a mean speed of 93.4 km/day (95% CIs: 87.6–99.4), whereas the SLD estimate was >3-fold lower at 27.3 km/day. To confirm the scale-insensitivity of the CTSD estimate for these data, versus the scale-sensitivity of SLD, we

repeat the analysis on the more finely sampled GPS data collected over the same period.

The first steps are to import the data, assign the RMS UERE, and check for outliers.

```
gps_data <- pelican[[2]]

#Assign a 10m UERE
uere(gps_data) <- 10

#Re-check for any remaining outliers
par(mfrow=c(1,3))

plot(DATA,
     error = 2,
     level.UD = 0.50)

OUTLIERS <- outlie(DATA)

plot(OUTLIERS)
```

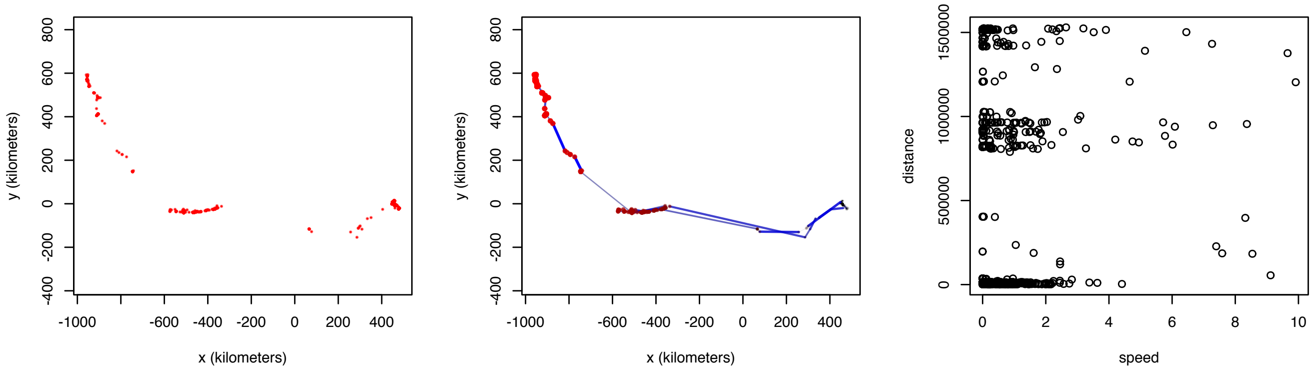

Figure 13: From left to right, the GPS positional observations returned by the `outlie()` function, and the speed and distance estimates returned by the `outlie()` function.

As with the ARGOS data, there is no clear evidence that any of these data are outliers, so we proceed with the model fitting and estimation steps.

```
#Fit and select the movement models
GUESS <- ctmm.guess(gps_data,
                    interactive = FALSE,
                    CTMM = ctmm(tau = c(Inf,
```

```
                                     2 %#% 'hours'),
                            range = FALSE))

GUESS$error <- TRUE

GPS_FIT <- ctmm.select(gps_data,
                       GUESS)

summary(GPS_FIT)

$name
[1] "IOU anisotropic error"

$DOF
    mean     area    speed
   0.000    0.000 1180.545

$CI
                              low        est       high
τ[velocity] (hours)      1.561744   1.720164   1.894655
speed (kilometers/day) 152.802686 157.289429 161.774421

#Estimate speed
gps_speed <- speed(gps_data, GPS_FIT, cores = -1)

                            low      est     high
speed (kilometers/day) 90.92524 93.96206 97.02328

GPS_SPEEDS <- speeds(gps_data,
                       GPS_FIT)

head(GPS_SPEEDS)

        low      est     high          t           timestamp
1 0.1885202 1.136441 2.506196 1513296033 2017-12-15 00:00:33
2 0.1905690 1.139954 2.509741 1513303216 2017-12-15 02:00:16
3 1.5093224 3.041988 4.822413 1513339234 2017-12-15 12:00:34
4 0.6270016 1.821023 3.313038 1513346433 2017-12-15 14:00:33
5 0.1815883 1.073224 2.356639 1513353659 2017-12-15 16:00:59
6 0.6555744 1.954969 3.587240 1513368011 2017-12-15 20:00:11


#The SLD Speed (in km/day)
#First get the duration of the sampling period (in days)
DURATION <- (tail(gps_data$t,1) - head(gps_data$t,1))/86400
```

```
#The SLD speed (distance/time)
(tot.dist(gps_data)/1000)/DURATION
```

```
39.77388
```

From these GPS data, we see that the CTSD estimate of 94.0 km/day (95%CIs 90.9–97.0) is nearly identical to the CTSD estimate on the ARGOS data (93.4 km/day; 95% CIs: 87.6–99.4), though with narrower confidence intervals on the more finely sampled GPS data. In contrast, the 39.8 km/day speed estimated via SLD on the GPS data is drastically greater than the 27.3 km.day estimated on the more coarsely sampled ARGOS data. Collectively these results confirm the scale-insensitivity and well-behaved confidence intervals of CTSD, as opposed to the scale-sensitivity of SLD. They further reveal that the primary source of bias in both the GPS and ARGOS data is tortuosity induced, a data regime where model-smoothing is unlikely to provide any major benefits to SLD estimation (see simulation results in the main text).

References

[1] Kays R, Hirsch BT. Data from: Stink or swim: techniques to meet the challenges for the study and conservation of small critters that hide, swim or climb and may otherwise make themselves unpleasant. Movebank Data Repository. 2015;.

[2] Calabrese JM, Fleming CH, Gurarie E. ctmm: an R package for analyzing animal relocation data as a continuous-time stochastic process. Methods in Ecology and Evolution. 2016 Sep;7(9):1124–1132.

[3] Hofmann-Wellenhof B, Lichtenegger H, Collins J. Global positioning system: theory and practice. Springer Science & Business Media; 2012.
