# Supplementary material for: Scale-insensitive estimation of speed and distance traveled from animal tracking data
Source: Mov Ecol. 2019 Nov 15;7:35. doi: 10.1186/s40462-019-0177-1 (PMC6858693; doi:10.1186/s40462-019-0177-1)
Supplement: Supplementary file 3 — Additional file 3 Technical details. Details on estimating the mean speed and root mean square (RMS) speed from either a time-averaged stationary Gaussian stochastic process or from instantaneous Kriged velocity estimates, and how we translate point estimates and standard errors into non-standard confidence intervals. [file 40462_2019_177_MOESM3_ESM.pdf]

Additional file S3 — Technical details

In this appendix we provide details on estimating the mean speed and root mean square (RMS) speed from either a time-averaged stationary Gaussian stochastic process or from instantaneous Kriged velocity estimates. For the following calculations we will assume the velocity $\mathbf{v}$ to be normally distributed with mean $\boldsymbol{\mu}$ and covariance $\boldsymbol{\sigma}$. The Gaussian RMS speed can be calculated very quickly from the fitted movement model's parameter estimates, but is not generally proportional to the distance travelled. The approach described in the main text represents the mean speed, which is conditioned off the data, and is more accurate when the data are not fully specified by the Gaussian model.

RMS speed

The RMS speed is easily related to the velocity variance

$$v_{\mathrm{RMS}} = \sqrt{\lim_{T\to\infty} \frac{1}{T} \int_{-T}^{+T} dt \, \langle \mathbf{v}(t)^2 \rangle} \,, \tag{1}$$

$$= \sqrt{\lim_{T\to\infty} \frac{1}{T} \int_{-T}^{+T} dt \, \langle \mathrm{tr}\, \mathbf{v}(t)\, \mathbf{v}(t)^{\mathrm{T}} \rangle} \,, \tag{2}$$

$$= \sqrt{\lim_{T\to\infty} \frac{1}{T} \int_{-T}^{+T} dt \, (\boldsymbol{\mu}(t)^2 + \mathrm{tr}\, \boldsymbol{\sigma}(t))} \,, \tag{3}$$

where for a stationary process, $v_{\mathrm{RMS}} = \sqrt{\mathrm{tr}\, \boldsymbol{\sigma}}$, or $v_{\mathrm{RMS}}(t) = \sqrt{\boldsymbol{\mu}(t)^2 + \mathrm{tr}\, \boldsymbol{\sigma}(t)}$ instantaneously.

Mean speed

The mean speed is derived from the mean absolute deviation $\langle |\mathbf{v}| \rangle$, which is difficult to calculate in general. First we will derive the mean speed under the assumption of $\boldsymbol{\mu} = \mathbf{0}$, which is sufficient for the time-average of a stationary process. Next we will derive the mean speed for a symmetric covariance matrix $\boldsymbol{\sigma}$. Finally, we will combine these exact results into an approximate formula for the general case.

Mean speed: zero mean

If $\boldsymbol{\mu} = \mathbf{0}$, then in two dimensions the mean speed is determined by the integral

$$\langle|\mathbf{v}|\rangle = \iint d^2\mathbf{v}\, \sqrt{\mathbf{v}^2}\, \frac{1}{\sqrt{\det 2\pi\boldsymbol{\sigma}}}\, e^{-\frac{1}{2}\mathbf{v}^{\mathrm{T}}\boldsymbol{\sigma}^{-1}\mathbf{v}}\,, \tag{4}$$

$$= \frac{1}{2\pi} \iint d^2\mathbf{v}\, \sqrt{\mathbf{v}^{\mathrm{T}}\boldsymbol{\sigma}\,\mathbf{v}}\, e^{-\frac{1}{2}\mathbf{v}^2}\,, \tag{5}$$

$$= \frac{1}{2\pi} \int_0^{2\pi} d\theta\, \sqrt{\mathbf{u}(\theta)^{\mathrm{T}}\boldsymbol{\sigma}\,\mathbf{u}(\theta)} \int_0^{\infty} dv\, v^2\, e^{-\frac{1}{2}v^2}\,, \tag{6}$$

$$= \sqrt{\frac{\pi}{2}}\, \frac{1}{2\pi} \int_0^{2\pi} d\theta\, \sqrt{\mathbf{u}(\theta)^{\mathrm{T}}\boldsymbol{\sigma}\,\mathbf{u}(\theta)}\,, \tag{7}$$

where $\mathbf{u}(\theta) = (\cos\theta, \sin\theta)$ is the unit vector. This relation can be simplified by rotating our polar coordinate system so that $\theta = 0$ occurs when $\mathbf{u}(\theta)$ is parallel with one of the eigen-vectors of the velocity covariance. Let $\sigma_\pm$ represent the two eigen-values of $\boldsymbol{\sigma}$, where $\sigma_+ \geq \sigma_-$. The mean speed integral then reduces to

$$\langle|\mathbf{v}|\rangle = \sqrt{\frac{\pi}{2}}\, \frac{1}{2\pi} \int_0^{2\pi} d\theta\, \sqrt{\sigma_+ \cos^2\theta + \sigma_- \sin^2\theta}\,, \tag{8}$$

$$= \sqrt{\frac{\pi}{2}}\, \frac{1}{2\pi} \int_0^{2\pi} d\theta\, \sqrt{\sigma_+ - (\sigma_+ {-} \sigma_-)\sin^2\theta}\,, \tag{9}$$

$$= \sqrt{\frac{2}{\pi}} \sqrt{\sigma_+} \int_0^{\frac{\pi}{2}} d\theta\, \sqrt{1 - \frac{\sigma_+ {-} \sigma_-}{\sigma_+}\sin^2\theta}\,, \tag{10}$$

$$= \sqrt{\frac{2}{\pi}} \sqrt{\sigma_+}\, E\!\left(\frac{\sigma_+ {-} \sigma_-}{\sigma_+}\right). \tag{11}$$

where $E(k)$ is the complete elliptic integral of the second kind. In the isotropic case where $\sigma_\pm = \sigma_0$ and $E(0) = \pi/2$, the mean speed reduces to $\sqrt{\pi/2\,\sigma_0}$.

Mean speed: zero eccentricity

If $\boldsymbol{\sigma} = \sigma_0\,\mathbf{I}$, then the two-dimensional mean speed is given by the integral

$$\langle|\mathbf{v}|\rangle = \iint d^2\mathbf{v}\, \sqrt{\mathbf{v}^2}\, \frac{1}{2\pi\sigma_0}\, e^{-\frac{1}{2\sigma_0}(\mathbf{v}-\boldsymbol{\mu})^2}\,, \tag{12}$$

$$= \frac{1}{2\pi\sigma_0} \int_0^{\infty} dv\, v^2\, e^{-\frac{v^2+\mu^2}{2\sigma_0}} \int_0^{2\pi} d\theta\, e^{+\frac{\mu v}{\sigma_0}\cos(\theta)}\,, \tag{13}$$

$$= \int_0^{\infty} dv\, \frac{v^2}{\sigma_0}\, e^{-\frac{v^2+\mu^2}{2\sigma_0}}\, I_0\!\left(\frac{\mu v}{\sigma_0}\right), \tag{14}$$

where $I_m(z)$ the mth order modified Bessel function of the first kind. The remaining integral resolves to

$$\langle|\mathbf{v}|\rangle = \sqrt{\frac{\pi}{2}\sigma_0}\, I_0\!\left(\frac{\mu^2}{2\sigma_0}\right) e^{-\frac{\mu^2}{2\sigma_0}} + \mu\sqrt{\frac{\pi}{2}}\sqrt{\frac{\mu^2}{2\sigma_0}}\left(I_0\!\left(\frac{\mu^2}{2\sigma_0}\right) + I_1\!\left(\frac{\mu^2}{2\sigma_0}\right)\right) e^{-\frac{\mu^2}{2\sigma_0}} \,. \tag{15}$$

Mean speed: combined approximation

To construct a generally applicable approximation, we compare the combined limits of zero mean and eccentricity, which results in a mean speed of $\sqrt{\pi/2\,\sigma_0}$. Relation (11) generalizes this result to non-zero eccentricity, while relation (15) generalizes this result to non-zero mean. In (15), one can identify the mean-zero limit as a factor of the first term, while the second term contains the large mean limit. Therefore we combine the two results directly to obtain the approximate relation

$$\langle|\mathbf{v}|\rangle \approx \sqrt{\frac{2}{\pi}}\sqrt{\sigma_+}\, E\!\left(\frac{\sigma_+ - \sigma_-}{\sigma_+}\right) I_0\!\left(\frac{\mu^2}{2\sigma_0}\right) e^{-\frac{\mu^2}{2\sigma_0}} + \mu\sqrt{\frac{\pi}{2}}\sqrt{\frac{\mu^2}{2\sigma_0}}\left(I_0\!\left(\frac{\mu^2}{2\sigma_0}\right) + I_1\!\left(\frac{\mu^2}{2\sigma_0}\right)\right) e^{-\frac{\mu^2}{2\sigma_0}} \,. \tag{16}$$

where $\sigma_0 = (\sigma_+ + \sigma_-)/2$. This result is exact in three limits: $\mu^2 \to 0$, $\mu \to \infty$, and $\sigma_+ \to \sigma_-$.

χ^2 and χ confidence intervals

In this appendix, we detail how we translate point estimates and standard errors into non-standard confidence intervals, which can be more appropriate than normal confidence intervals if the sampling distribution more resembles another. All of these confidence intervals obey the central limit theorem and share the same first two moments or cumulants.

χ^2 confidence intervals

If a statistic X is proportionally χ^2_k, then its mean and variance obey the relation

$$\frac{\mathrm{VAR}[X]}{\langle X\rangle^2} = \frac{2}{k} \,, \tag{17}$$

and so the degrees of freedom are given by

$$k = \frac{2\,\langle X\rangle^2}{\mathrm{VAR}[X]} \,. \tag{18}$$

We use CIs derived from this distribution on square speed estimates, because they are exact in some cases then.

χ confidence intervals

If a statistic X is proportionally χ^1_k, then its mean and variance obey the relation

$$\underbrace{\frac{\langle X\rangle^2}{\mathrm{VAR}[X]+\langle X\rangle^2}}_{R} = \underbrace{\frac{2\pi}{k}\mathrm{B}\left(\frac{k}{2},\frac{1}{2}\right)^{-2}}_{f(k)}, \tag{19}$$

where B denotes the beta function. We use CIs derived from this distribution on mean speed estimates, because they are exact in some cases. To solve $k = f^{-1}(R)$, we expand about a numerical estimate k_i and update to get

$$R = f(k_i) + f'(k_i)\,(k-k_i) + \mathcal{O}\big((k-k_i)^2\big)\,, \qquad k_{i+1} = k_i + \frac{R-f(k_i)}{f'(k_i)}\,, \tag{20}$$

$$f'(k) = f(k)\left(1 - \frac{1}{k} + \psi\left(\frac{k+1}{2}\right) - \psi\left(\frac{k}{2}\right)\right), \tag{21}$$

where $\psi(z) = \psi_0(z)$ is the digamma function. For the initial numerical estimate, we use the asymptotic expansion of the ratio of the variance to the square mean

$$\frac{\mathrm{VAR}[X]}{\langle X\rangle^2} = R^{-1} - 1 = \frac{1}{2k} + \mathcal{O}\big(k^{-2}\big)\,, \qquad k_0 = \frac{\langle X\rangle^2}{2\,\mathrm{VAR}[X]} = \frac{1}{2}\frac{1}{R^{-1}-1}\,, \tag{22}$$

which is analogous to the χ^2 relation and well behaved.
