# Supplementary material for: Scale-insensitive estimation of speed and distance traveled from animal tracking data
Source: Mov Ecol. 2019 Nov 15;7:35. doi: 10.1186/s40462-019-0177-1 (PMC6858693; doi:10.1186/s40462-019-0177-1)
Supplement: Supplementary file 4 — Additional file 4 R script for reproducing the simulations. [file 40462_2019_177_MOESM4_ESM.pdf]

Additional file S4 — R script for reproducing the simulations comparing CTSD to SLD

In this appendix we provide the R script required to reproduce the SLD and CTSD comparison simulations.

Sampling frequency

As described in the main text, our first set of simulations tested how variation in sampling frequencies influenced estimates. We set the position and velocity autocorrelation timescales to 1 day, and 1 hour respectively, and simulated a fine scale trajectory from this model, sampled for 10 days at a frequency of 4096 locations/day. This fine-scale, error-free trajectory was used to estimate the true distance travelled. After determining the truth, mean-zero Gaussian error with a standard deviation of 10m was added to each location. Using the data with added measurement error, we estimated the total distance traveled using both conventional SLD and model-smoothed SLD, in addition to CTSD estimation, and compared each of these estimates to the truth. We then thinned down the fine-scale trajectory by removing every second location, and repeated the model fitting and estimation process. This thinning and re-estimation was repeated to generate increasingly coarse data with sampling frequencies that ranged from the full resolution of 4096 locations/day, down to 8 locations/day in a halving series. The simulation, estimation, and iterative thinning process was then repeated 100 times, and results were compared across simulation runs.

R Script

```
#Load in the requisite packages into the root environment

library(ctmm)
library(foreach)
library(doParallel)

#Build any other necessary functions

############################
#A function for calculating the SLD between supplied locations
tot.dist <- function(data){

  distance <- list()

  for(i in 2:nrow(data)){

      distance[[i]] <- sqrt((data$x[i] - data$x[i-1])^2 + (data$y[i] -
         data$y[i-1])^2)
  }
```

```
  sum(unlist(distance))
}

########################################################################
############### Set up the sampling frequency simulation #############
########################################################################

#1 day in seconds
ds <- 86400

#Spatial variance in m^2
sig <- 100000

#Specify an OUF model for simulation
#Here tau velocity is 1/24 of tau position (i.e., 1 hour)
mod1 <- ctmm(tau=c(ds,ds/24),
             isotropic=TRUE,
             sigma=sig,
             mu=c(0,0))

nd <- 10 #sampling duration in days
pd <- 4096 #number of samples per day

#sampling times
st <- 1:(nd*pd)*(ds/pd)

#The proportion of data to thin
thin <- c(8, 16, 32, 64, 128, 256, 512, 1024, 2048, 4096)

#The number of replicates
nReps <- 100

res <- list()

#Loop over the thinning vector
for(i in 1:length(thin)){

  #Create an empty list for storing results
  x <- list()

  for(j in 1:nReps){

    #Print out a progress report
    cat("Starting iteration ", j, "for thinning factor", thin[i], "\n")

    message("Generating the simulated data")
```

```
#Simulate some data from the model
sim1 <- simulate(mod1,t=st)

#Add normally distributed error to the data
error1 <- rnorm(nrow(sim1), mean = 0, sd = 10)
error2 <- rnorm(nrow(sim1), mean = 0, sd = 10)

sim.error <- sim1

sim.error[c(2,3)] <- sim.error[c(2,3)] + c(error1, error2)

#Generate a duplicate dataset without UERE information
#This is used in the joint estimation for model-smoothed SLD
sim.error.smoothing <- sim.error

#Define the UERE for CTSD with a correctly specified error model
sim.error$VAR.xy <- 100

#Use the speed function on the unthinned data, and the true model
   to get the true speed
true_dist <- ctmm::speed(sim1,
                         mod1,
                         cores = -1,
                         prior = FALSE,
                         units = FALSE)

################################################################
#Thin the data according to the thinning vector defined above
rows.thinned <- seq(1, nrow(sim.error), by=round(pd/thin[i]))

#Thinned data with UERE information
data.thinned <- sim.error[rows.thinned,]

#Thinned data without UERE information
data.smoothing <- sim.error.smoothing[rows.thinned,]

#Store the sampling frequency of the thinned data (fixes/day)
freq <- nrow(data.thinned)/nd

################################################################
#The distance estimated by SLD
tot_dist <- tot.dist(data.thinned)

################################################################
#The speed estimated by the CTSD method
```

```
#NOTE, this first requires fitting a movement model to the data

message("Fitting the movement model with calibrated errors")
sim.mod <- ctmm.fit(data.thinned,
                    CTMM = ctmm(error=TRUE,
                                tau=c(ds,ds/24),
                                isotropic=TRUE,
                                sigma=sig,
                                mu=c(0,0)))

     ctmm_dist <- speed(data.thinned,
                        sim.mod,
                        cores = -1,
                        units = FALSE)

##############################################################
#The model-smoothed SLD
message("Jointly estimating the movement and error models")

#First jointly estimate the process and error variances
    smoothing.mod <- ctmm.fit(data.smoothing,
                              CTMM = ctmm(error=TRUE,
                                          tau=c(ds,ds/24),
                                          isotropic=TRUE,
                                          sigma=sig,
                                          mu=c(0,0)))

#Smooth the data based on the fitted model
data.smoothed <- predict(data.smoothing,
                         smoothing.mod,
                         t= data.smoothing$t)

#Estimate the SLD based on the smoothed data
smoothed.SLD <- tot.dist(data.smoothed)

#Print out another progress report
cat("Finished iteration ", j, "for thinning factor", thin[i], "\n")

##############################################################
#Store the vector of results in the list created above
x[[j]] <- c(thin[i], #The thinning factor
            freq, #The thinned frequency (fixes/day)
            true_dist[2]*(nd %#% "days"), #The 'true' distance
            tot_dist, #The distance calculated by SLD
            smoothed.SLD, #The smoothed SLD
            ctmm_dist[2]*(nd %#% "days"), #The ctsd estimate
```

```
                  ctmm_dist[1]*(nd %#% "days"), #Min CI
                  ctmm_dist[3]*(nd %#% "days") #Max CI
    )
  }

  #Unlist the results for this parameterisation
  x <- do.call(rbind, x)

  #And store them in a list
  res[[i]] <- x



  #Save the results as they come out (in case of crashes)
  save(res, file = "Speed_Sims_Smoothing.rda")


  #Spit out an indicator of progress
  print(thin[i])
}

res <- do.call(rbind, res)

res <- data.frame("thining.fraction" = res[,1],
                  "thinned.frequency" = res[,2],
                  "true.distance" = res[,3],
                  "SLD" = res[,4],
                  "smoothed.SLD" = res[,5],
                  "CTSD.ML" = res[,6],
                  "CTSD.min" = res[,7],
                  "CTSD.max" = res[,8])

save(res, file = "Speed_Sims_Smoothing.rda")
```

Irregular sampling

In the second set of simulations, we tested the performance of conventional SLD, model-smoothed SLD and CTSD on data with irregular sampling, where we mimicked the effect of random, sporadic data loss. We set the position and velocity autocorrelation timescales to 1 day, and 1 hour respectively, and simulated a trajectory sampled for 10 days at a constant frequency of 64 locations/day. Again, after determining the truth, mean-zero Gaussian error with a standard deviation of 10m was added to each location. We then randomly dropped a percentage of the collected locations (ranging from 0% — i.e., no data loss — to 70%, and increasing by 5% increments), where increasing the percentage of data loss resulted in increasingly irregular data. Using the irregularly thinned data with error, we estimated the total distance traveled using conventional SLD, model-smoothed SLD,

and CTSD estimation, and compared these estimates to the truth.

R Script

```
######################################################################
############### Set up the irregular sampling simulation #############
######################################################################

#1 day in seconds
ds <- 86400

#Spatial variance in m^2
sig <- 100000

#Specify an OUF model for simulation
mod1 <- ctmm(tau=c(ds,ds/24), isotropic=TRUE, sigma=sig, mu=c(0,0))

nd <- 10 #sampling duration in days
pd <- 64 #number of samples per day

#sampling times
st <- 1:(nd*pd)*(ds/pd)

#The proportion of data to thin
loss <- c(1, 0.95, 0.90, 0.80, 0.75, 0.70, 0.65, 0.60, 0.55, 0.50,
   0.45, 0.40, 0.35, 0.30)

#The number of replicates
nReps <- 100

res <- list()

#Loop over the vector defining the proportion of data to randomly thin
for(i in 1:length(loss)){

  #Create an empty list for storing results
  x <- list()

  for(j in 1:nReps){

    cat("Starting iteration ", j, "for a proportional loss ", (1-loss[i
       ])*100, "% \n")

    message("Generating the simulated data")

    #Simulate some data from the model
```

```
sim1 <- simulate(mod1,t=st)

#Add normally distributed error to the data
error1 <- rnorm(nrow(sim1), mean = 0, sd = 10)
error2 <- rnorm(nrow(sim1), mean = 0, sd = 10)

sim.error <- sim1

sim.error[c(2,3)] <- sim.error[c(2,3)] + c(error1, error2)

#Generate a duplicate dataset without UERE information
#This is used in the joint estimation for model-smoothed SLD
data.smoothing <- sim.error

#Assign the 10m error as the UERE
sim.error$VAR.xy <- 100

#Use the speed function on the unthinned data, and the original
   model to get the true speed
true_dist <- ctmm::speed(sim1,
                         mod1,
                         prior = FALSE,
                         units = FALSE)

################################################################
#Thin the data according to the proportional loss vector defined
   above
ST <- sort(sample(st, size = (length(st)*loss[i])))

#Thinned data with UERE information
data.thinned <- sim.error[which(data.smoothing$t %in% ST),]

#Thinned data withou UERE information
data.smoothing <- data.smoothing[which(data.smoothing$t %in% ST),]

#Store the sampling frequency of the thinned data (fixes/day)
freq <- nrow(data.thinned)/nd

################################################################
#The speed estimated by SLD
tot_dist <- tot.dist(data.thinned)

################################################################
#The speed estimated by the CTSD method
#NOTE, this first requires fitting a movement model to the data
```

```
  message("Fitting the movement model with calibrated errors")
  sim.mod <- ctmm.fit(data.thinned,
                      CTMM = ctmm(error=TRUE,
                                  tau=c(ds,ds/24),
                                  isotropic=TRUE,
                                  sigma=sig,
                                  mu=c(0,0)))

  ctmm_dist <- speed(data.thinned,
                     sim.mod,
                     cores = -1,
                     units = FALSE)

  ################################################################
  #The model -smoothed SLD
  message("Jointly estimating the movement and error models")

  #The smoothed SLD
  smoothing.mod <- ctmm.fit(data.smoothing,
                            CTMM = ctmm(error=TRUE,
                                        tau=c(ds,ds/24),
                                        isotropic=TRUE,
                                        sigma=sig,
                                        mu=c(0,0)))

  data.smoothed <- predict(data.smoothing,
                           smoothing.mod,
                           t= data.smoothing$t)

  smoothed.SLD <- tot.dist(data.smoothed)

  cat("Finished iteration ", j, "for a proportional loss ", (1-loss[i
     ])*100, "% \n")

  ################################################################
  #Store the vector of results in the list created above
  x[[j]] <- c(loss[i], #The thinning factor
              freq, #The thinned frequency (fixes/day)
              true_dist[2]*(nd*ds), #The 'true' distance
              tot_dist, #The distance calculated by step length
              smoothed.SLD, #The smoothed SLD
              ctmm_dist[2]*(nd*ds), #The bootstrapped speed estimate
              ctmm_dist[1]*(nd*ds), #Min CI
              ctmm_dist[3]*(nd*ds) #Max CI
  )
} #close the 'iterations' loop
```

```r
  #Unlist the results for this parameterisation
  x <- do.call(rbind, x)

  #And store them in a list
  res[[i]] <- x

  #Save the results as they come out (in case of crashes)
  save(res, file = "IrregularSampling_Sims_Smoothing.rda")

  #Spit out an indicator of progress
  print(loss[i])
} #Close the outer loop


res <- do.call(rbind, res)

res <- data.frame("data.loss" = res[,1],
                  "thinned.frequency" = res[,2],
                  "true.distance" = res[,3],
                  "SLD" = res[,4],
                  "smoothed.SLD" = res[,5]),
                  "CTSD.ML" = res[,6],
                  "CTSD.min" = res[,7],
                  "CTSD.max" = res[,8])

save(res, file = "IrregularSampling_Sims_Smoothing.rda")
```

Movement tortuosity

In the third set of simulations, we tested how variation in the tortuosity of an individual's movement influenced estimates. Here, we simulated a trajectory sampled for 10 days at a constant frequency of 64 locations/day. We set the position autocorrelation timescales to 1 day, but manipulated the velocity autocorrelation timescale (ranging from 11.25 minutes to 1 day in a doubling series), where increasing the duration of velocity autocorrelation generates movement that is decreasingly tortuous (i.e., more linear, [6]). After determining the truth, mean-zero Gaussian error with a standard deviation of 10m was added to each location. The total distance traveled was then estimated using SLD, model-smoothed SLD and CTSD as described above, and these estimates were compared to the truth.

R Script

```r
######################################################################
##########         Simulations varying tortuosity        #############
######################################################################
```

```
#1 day in seconds
ds <- 86400

#Spatial variance in m^2
sig <- 100000

nd <- 10 #sampling duration in days
pd <- 64 #number of samples per day

#sampling times
st <- 1:(nd*pd)*(ds/pd)

#The vector of tau_v values to simulate
tau_v <- c(1, 2, 4, 8, 16, 32, 64, 128)

#The number of iterations
nReps <- 100

res <- list()

#Loop over the tau_v values to simulate from
for(i in 1:length(tau_v)){

  x <- list()

  for(j in 1:nReps){

    cat("Starting iteration ", j, "for a tau_v of ", (ds/tau_v[i])/60,
       "min \n")
    message("Generating the simulated data")

    #Define the movement model
    mod1 <- ctmm(tau=c(ds,
                       (ds-1)/tau_v[i]),
                 isotropic=TRUE,
                 sigma=sig,
                 mu=c(0,0))

    #Simulate some data from the model
    sim1 <- simulate(mod1,t=st)

    #Add normally distributed error to the data
    error1 <- rnorm(nrow(sim1), mean = 0, sd = 10)
    error2 <- rnorm(nrow(sim1), mean = 0, sd = 10)
```

```
sim.error <- sim1
sim.error[c(2,3)] <- sim.error[c(2,3)] + c(error1, error2)

#Generate a duplicate dataset without UERE information
#This is used in the joint estimation for model-smoothed SLD
data.smoothing <- sim.error

#Assign the 10m error as the UERE
sim.error$VAR.xy <- 100

#Use the speed function on the data without error, and the true
   model to get the true speed
true_dist <- ctmm::speed(sim1,
                         mod1,
                         prior = FALSE,
                         units = FALSE)

################################################################
#The speed estimated by SLD
tot_dist <- tot.dist(sim.error)

################################################################
#The speed estimated by the CTSD method
#NOTE, this first requires fitting a movement model to the data

message("Fitting the movement model with calibrated errors")
sim.mod <- ctmm.fit(sim.error,
                     CTMM = ctmm(error=TRUE,
                                 tau=c(ds,(ds-1)/thin[i]),
                                 isotropic=TRUE,
                                 sigma=sig, mu=c(0,0)))

ctmm_dist <- speed(data.thinned,
                   sim.mod,
                   cores = -1,
                   units = FALSE)

################################################################
#The model-smoothed SLD
message("Jointly estimating the movement and error models")

smoothing.mod <- ctmm.fit(data.smoothing,
                          CTMM = ctmm(error=TRUE,
                                      tau=c(ds,
                                            (ds-1)/thin[i]),
                                      isotropic=TRUE,
```

```
                                                   sigma=sig,
                                                   mu=c(0,0)))

    data.smoothed <- predict(data.smoothing,
                             smoothing.mod,
                             t= data.smoothing$t)

    smoothed.SLD <- tot.dist(data.smoothed)

    #Print out another progress report
    cat("Finished iteration ", j, "for a tau_v of ", (ds/tau_v[i])/60,
       "min \n")

    ################################################################
    #Store the vector of results in the list created above
    x[[j]] <- c(tau_v[i], #The thinning factor
                true_dist[2]*(nd*ds), #The 'true' distance
                tot_dist, #The distance calculated by SLD
                smoothed.SLD, #The model-smoothed SLD
                ctmm_dist[2]*(nd*ds), #The CTSD estimate
                ctmm_dist[1]*(nd*ds), #Min CI
                ctmm_dist[3]*(nd*ds) #Max CI
    )
  } #Close the 'iterations' loop

  #Unlist the results for that parameterisation
  x <- do.call(rbind, x)

  #And store them in a list
  res[[i]] <- x

  #Save the results as they come out (in case of crashes)
  save(res, file = "Tortuosity_Sims_Smoothing.rda")

  #Spit out an indicator of progress
  print(thin[i])

} #Close the outer loop

res <- do.call(rbind, res)
res <- data.frame("tau_v" = res[,1],
                  "true.distance" = res[,2],
                  "SLD" = res[,3],
                  "smoothed.SLD" = res[,4],
                  "CTSD.ML" = res[,5],
                  "CTSD.min" = res[,6],
```

```
                    "CTSD.max" = res[,7])

save(res, file = "Tortuosity_Sims_Smoothing.rda")
```

Location error

In the final set of simulations, we tested how variation in the amount of measurement error influenced estimates. Here, we simulated 100 trajectories, sampled for 10 days at a fixed frequency of 64 locations/day. We set the position and velocity autocorrelation timescales to 1 day, and 1 hour respectively. After simulation, we again added mean-zero Gaussian error to each location, but here manipulated the standard deviation of the distribution (ranging from 0, i.e., no error, to 51.2 meters, in a doubling series of the minimal 0.1 m error).

R Script

```
######################################################################
##########      Simulations varying error magnitude      #############
######################################################################

#1 day in seconds
ds <- 86400

#Spatial variance in m^2
sig <- 100000

nd <- 10 #sampling duration in days
pd <- 256 #number of samples per day

#sampling times
st <- 1:(nd*pd)*(ds/pd)

#The SD of the Gaussian error distribution
error <- c(0, 0.1, 0.2, 0.4, 0.8, 1.6, 3.2, 6.4, 12.8, 25.6, 51.2)

#The number of iterations
nReps <- 100

#Empty for storing results
res <- list()

#Loop over durations (days) or sampling frequencies (samp), as
   appropriate
for(i in 1:length(error)){

  x <- list()
```

```
for(j in 1:nReps){
  cat("Starting iteration ", j, "for an error of ", error[i], "m \n")
  message("Generating the simulated data")

  #Define the movement model
  mod1 <- ctmm(tau=c(ds,ds/24), isotropic=TRUE, sigma=sig, mu=c(0,0))

  #Simulate some data from the model
  sim1 <- simulate(mod1,t=st)

  #Add normally distributed error to the data with the desired SD
  error1 <- rnorm(nrow(sim1), mean = 0, sd = error[i])
  error2 <- rnorm(nrow(sim1), mean = 0, sd = error[i])

  sim.error <- sim1
  sim.error[c(2,3)] <- sim.error[c(2,3)] + c(error1, error2)

  #Generate a duplicate dataset without UERE information
  #This is used in the joint estimation for model-smoothed SLD
  data.smoothing <- sim.error

  #Assign the appropriate UERE to the data
  sim.error$VAR.xy <- error[i]^2


  #Use the speed function on the data without error, and the original
      model to get the true speed
  true_dist <- ctmm::speed(sim1,
                           mod1,
                           prior = FALSE,
                           units = FALSE)

  ###########################################################
  #The speed estimated by SLD
  tot_dist <- tot.dist(sim.error)

  ###########################################################
  #The speed estimated by the CTSD method
  #NOTE, this first requires fitting a movement model to the data
  message("Fitting the movement model with calibrated errors")

  #Only turn error on when there has been error added to the data
  if(i == 1){
     ERROR <- FALSE
   } else{
```

```
   ERROR <- TRUE
  }

sim.mod <- ctmm.fit(sim.error,
                     CTMM = ctmm(error=ERROR,
                                 tau=c(ds,
                                       ds/24),
                                 isotropic=TRUE,
                                 sigma=sig,
                                 mu=c(0,0)))

ctmm_dist <- speed(sim.error,
                   sim.mod,
                   cores = -1,
                   units = FALSE)

################################################################
message("Jointly estimating the movement and error models")

#The model-smoothed SLD
smoothing.mod <- ctmm.fit(data.smoothing,
                          CTMM = ctmm(error=TRUE,
                                      tau=c(ds,
                                            ds/24),
                                      isotropic=TRUE,
                                      sigma=sig,
                                      mu=c(0,0)))


data.smoothed <- predict(data.smoothing,
                         smoothing.mod,
                         t= data.smoothing$t)


smoothed.SLD <- tot.dist(data.smoothed)

#Print out another progress report
cat("Finished iteration ", j, "for an error of ", error[i], "m \n")

#################################
#Vector of results to return
x[[j]] <- c(error[i], #The thinning factor
            true_dist[2]*(nd*ds), #The 'true' distance
            tot_dist, #The SLD
            smoothed.SLD, #The model-smoothed SLD
            ctmm_dist[2]*(nd*ds), #The CTSD estimate
```

```
                  ctmm_dist[1]*(nd*ds), #Min CI
                  ctmm_dist[3]*(nd*ds) #Max CI
    )

  } #Close the 'iterations' loop

  #And store them in a list
  res[[i]] <- x

  #Save the results as they come out (in case of crashes)
  save(res, file = "Speed_Error_Sims_Smoothing.rda")

  #Spit out an indicator of progress
  print(error[i])

} #Close the outer loop

res <- do.call(rbind, res)

res <- data.frame("error" = res[,1],
                  "true.distance" = res[,2],
                  "SLD" = res[,3],
                  "smoothed.SLD" = res[,4],
                  "CTSD.ML" = res[,5],
                  "CTSD.min" = res[,6],
                  "CTSD.max" = res[,7])

save(res, file = "Speed_Error_Sims_Smoothing.rda")
```

References

[1] Jonsen ID, Myers RA, James MC. Identifying leatherback turtle foraging behaviour from satellite telemetry using a switching state-space model. Marine Ecology Progress Series. 2007;337:255–264.

[2] Breed GA, Jonsen ID, Myers RA, Bowen WD, Leonard ML. Sex-specific, seasonal foraging tactics of adult grey seals (Halichoerus grypus) revealed by state–space analysis. Ecology. 2009;90(11):3209–3221.

[3] Cagnacci F, Boitani L, Powell RA, Boyce MS. Animal ecology meets GPS-based radiotelemetry: a perfect storm of opportunities and challenges. Philosophical Transactions of the Royal Society of London B: Biological Sciences. 2010;365(1550):2157–2162. Available from: http://rstb.royalsocietypublishing.org/content/365/1550/2157.

[4] Frair JL, Nielsen SE, Merrill EH, Lele SR, Boyce MS, Munro RHM, et al. Removing GPS collar bias in habitat selection studies. Journal of Applied Ecology. 2004;41(2):201–

212. Available from: https://besjournals.onlinelibrary.wiley.com/doi/abs/10.1111/j.0021-8901.2004.00902.x.

[5] Frair JL, Fieberg J, Hebblewhite M, Cagnacci F, DeCesare NJ, Pedrotti L. Resolving issues of imprecise and habitat-biased locations in ecological analyses using GPS telemetry data. Philosophical Transactions of the Royal Society of London B: Biological Sciences. 2010;365(1550):2187–2200. Available from: http://rstb.royalsocietypublishing.org/content/365/1550/2187.

[6] Fleming CH, Calabrese JM, Mueller T, Olson KA, Leimgruber P, Fagan WF. From fine-scale foraging to home ranges: A semivariance approach to identifying movement modes across spatiotemporal scales. The American Naturalist. 2014 May;183(5):E154–E167.
