# Supplementary material for: Scale-insensitive estimation of speed and distance traveled from animal tracking data
Source: Mov Ecol. 2019 Nov 15;7:35. doi: 10.1186/s40462-019-0177-1 (PMC6858693; doi:10.1186/s40462-019-0177-1)
Supplement: Supplementary file 5 — Additional file 5 CTSD and model misspecification. Evaluations of the performance of CTSD with misspecified models for scenarios that are likely to occur in real data. [file 40462_2019_177_MOESM5_ESM.pdf]

Additional file S5 — CTSD and model misspecification

As noted in the main text, the accuracy of our continuous-time speed and distance (CTSD) method will depend on how well the fitted model captures the structure of the empirical data. If the fitted model does not accurately capture key features in the data, then CTSD estimates will be prone to bias. This happens because the simulated trajectories used to generate the estimate are not fully representative of the path the animal actually travelled. In this appendix, we evaluate the performance of CTSD with a misspecified model for two specific scenarios that are likely to occur in real data: i) periodic border patrolling; and ii) central place foraging. In both cases the underlying movement process is non-stationary (i.e., the parameters of the movement model change over time), but we fit a stationary model that did not account for this non-stationarity.

Fitting a stationary model to non-stationary, periodic behavior

We first investigated the influence of model misspecification on CTSD estimation for simulated data emulating an animal that periodically patrols the outer boundary of their territory [1], but where this behavior was not accounted for in the fitted model.

To simulate these data, we defined a movement model with correlated positions and velocities, and a periodic mean [1]. This generates range-resident movement with a periodic pattern of space use within the home range, while all other movement parameters do not change over time. We set the position, and velocity autocorrelation timescales to 1 day and 1 hour respectively, and included a 1 day periodicity around the central location. We then simulated 10 days worth of data from this model, sampled at a 10 minute interval, and used this trajectory to calculate the true distance travelled. From this, we found that our simulated animal travelled a total of 65.9 km over the 10 day sampling period. After calculating the truth, we introduced mean-zero Gaussian error with a standard deviation of 10m to each location (Fig. 1).

We then continued with the `ctmm`-based CTSD workflow (see Fig. 3 in the main text), and proceeded to fit an Ornstein-Uhlenbeck Foraging (OUF) movement model [2] to the full simulated dataset, with error. Notably, this model does not account for the periodicity in the data. When compared to the semi-variogram (Fig. 2), although we found that the fitted model accurately captured features in the data at very short time-lags, at longer time-lags there were fairly substantial discrepancies between the data and the fitted model. Despite this, CTSD conditioned on the misspecified model was still capable of accurately estimating the instantaneous speeds (Fig. 3), and distance travelled (65.9 km; 95% CI: 65.8 – 66.9 km). As noted in the main text, asymptotic consistency does not require a correctly specified movement model, as long as the estimated covariance is 'compatible' with the true covariance [3].

Fitting a stationary model to a central place forager

We also investigated the influence of model misspecification on CTSD estimation for simulated data emulating central place foraging. Central place foraging is a common behavior for many species,

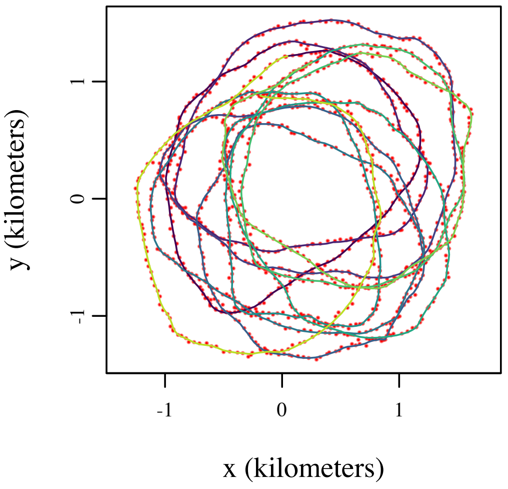

Figure 1: Simulated movement data for an animal that exhibited periodic border patrolling behavior. The colored trajectories depict the true path the simulated animal traveled (each color corresponds to one day), and the red points the sampled locations with 10m error.

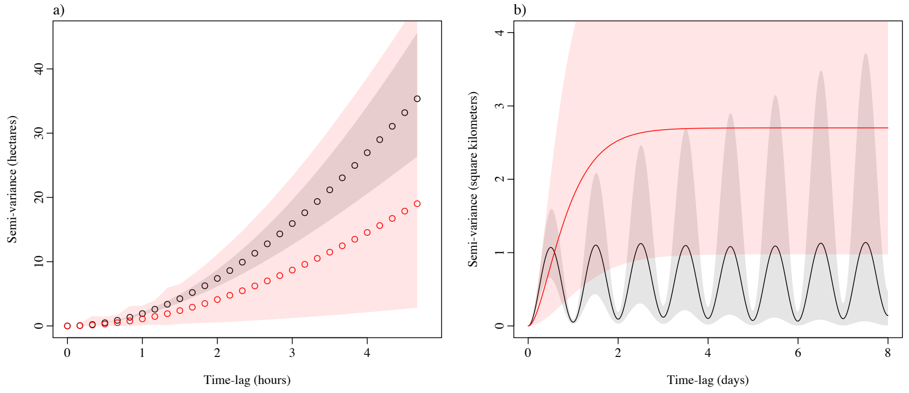

Figure 2: Semi-variogram of the simulated periodic data at a) short; and b) long time-lags. The black line and grey shading depict the semi-variance and 95% CIs, whereas the red line and shading depict the fitted movement model and 95% CIs of the model fit.

in which individuals alternate between active foraging bouts and periods of rest in a central place [typically a nest or burrow; 4; 5; 6]. When individuals alternate between dramatically different behavioral states (e.g., rest and search), then fitting one stationary model (i.e., a model for which the parameters do not change over time) to both behaviors will shoot down the middle, and tend to over-estimate speed during the rest period and under-estimate speed during the active period.

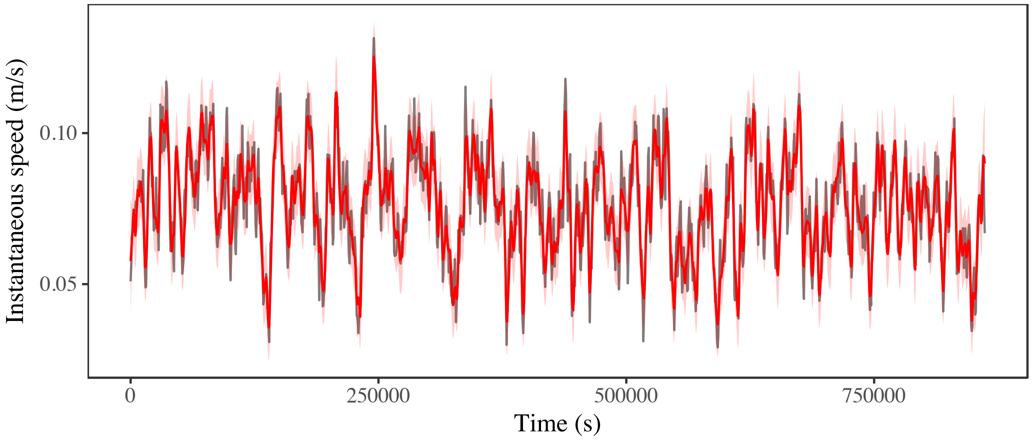

Figure 3: Instantaneous speed of the simulated periodic data over time. The gray line depicts the true instantaneous speed, whereas the red line and shading depict the estimates and 95% CIs.

To simulate these data, we defined an OUF model with position, and velocity autocorrelation timescales of 12 hours and 2 hours respectively. We then used this model to simulate a trajectory that started at $(x, y) = (0, 0)$, and returned to the same location 16 hours later. For the next 8 hours, the animal remained fixed at $(x, y) = (0, 0)$. This simulation process was repeated for 6 days, resulting in simulated data covering a total of 6 foraging trips, and 6 rest periods, sampled at a 5 minute interval. To calculate the truth, we quantified distance travelled for each of the fine-scale active periods separately, ignoring the inactive periods, and then summed these values. From this, we found that, over the 6 days, our simulated central place forager travelled a total of 6.51 km. After calculating the truth, we introduced mean-zero Gaussian error with a standard deviation of 10m to each location (Fig. 4).

We again fit an Ornstein-Uhlenbeck Foraging (OUF) movement model [2] to the full simulated dataset, with error. This is a stationary model that does not account for any change in movement behavior over time, such as the active/inactive pattern of these simulated central-place-foraging data. Comparing the fitted model to the semi-variogram, although we found that the model accurately captured the (time-averaged) fine-scale features in the data (Fig. 5a), there were some discrepancies at coarser time-lags (Fig. 5b). As a result of modeling a non-stationary process with a stationary model, the CTSD estimate was positively biased at 7.58 km (95% CI: 7.35 – 7.81 km; Fig. 6), with deceivingly narrow confidence intervals that did not provide coverage of the truth.

The approach of fitting a single stationary model to a non-stationary process clearly overestimated the total distance travelled. However, it is possible to correct for this model misspecification induced bias in a straightforward way, by breaking up the data according to the behavioral states. Here, for instance, this would involve splitting the 6 days of data into 12 individual datasets, each covering the individual foraging trips and rest periods, fitting a stationary movement model to

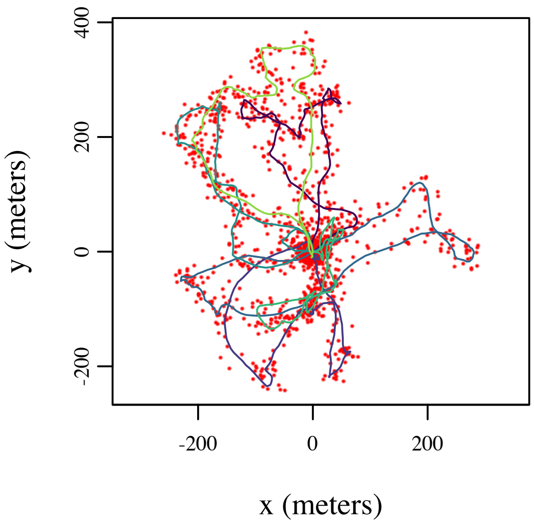

Figure 4: Simulated data for a central place forager. The colored trajectories depict the true foraging trajectories (each color corresponds to one trip), and the red points the sampled locations.

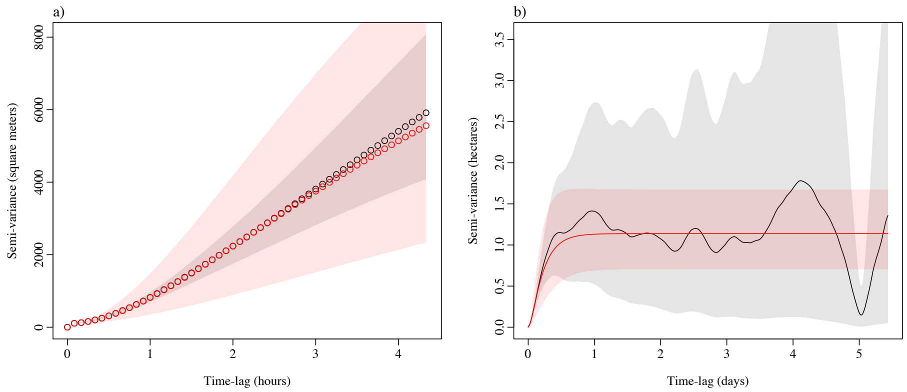

Figure 5: Semi-variogram of the simulated central place foraging data at a) short; and b) long time-lags. Note the 'nugget' in panel a), resulting from the measurement error.

each of the foraging trips individually, and then conditioning the CTSD estimates on these models. Doing this for our simulated central place foraging data, we found that there was minimal bias in each of the distance travelled estimates when stationary models were fit to data with single behavioural states. Furthermore, in all cases the 95% confidence intervals provided coverage of the truth (Table 1).

We recommend caution when working with non-stationary data. It should also be confirmed that all modeling assumptions have been satisfied, and that the resulting model fits are reasonable

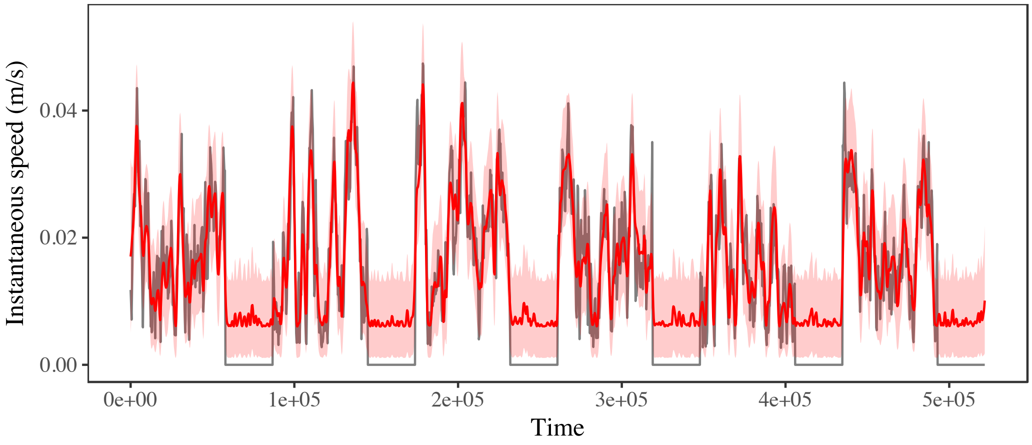

Figure 6: Instantaneous speed of the simulated central place foraging data over time. The gray line depicts the true instantaneous speed, whereas the red line and shading depict the estimates and 95% CIs. Note how the model does not account for the periods of inactivity, resulting in positively biased speed estimates.

prior to using the models to perform any conditional analyses.

Table 1: The true, and estimated distance travelled over each of the days, in km. Estimates were generated using continuous-time speed and distance estimation (CTSD).

	**Truth**	**CTSD ML**	**95% CIs**
**Day 1**	1.06	0.97	(1.08 – 1.20)
**Day 2**	1.07	1.18	(1.08 – 1.28)
**Day 3**	1.29	1.33	(1.28 – 1.39)
**Day 4**	1.09	1.12	(1.01 – 1.23)
**Day 5**	0.86	0.96	(0.84 – 1.07)
**Day 6**	1.13	1.14	(1.04 – 1.23)

References

[1] Péron G, Fleming CH, de Paula RC, Mitchell N, Strohbach M, Leimgruber P, et al. Periodic continuous-time movement models uncover behavioral changes of wild canids along anthropization gradients. Ecological Monographs. 2017 May;87(3):442–456.

[2] Fleming CH, Calabrese JM, Mueller T, Olson KA, Leimgruber P, Fagan WF. From Fine-Scale Foraging to Home Ranges: A Semivariance Approach to Identifying Movement Modes across Spatiotemporal Scales. The American Naturalist. 2014 May;183(5):E154–E167.

[3] Stein ML, Handcock MS. Some asymptotic properties of kriging when the covariance function is misspecified. Mathematical Geology. 1989;21(2):171–190.

[4] Orians GH. On the theory of central place foraging. Analysis of ecological systems. 1979;p. 157–177.

[5] Kacelnik A. Central place foraging in starlings (Sturnus vulgaris). I. Patch residence time. The Journal of Animal Ecology. 1984;p. 283–299.

[6] Olsson O, Brown JS, Helf KL. A guide to central place effects in foraging. Theoretical population biology. 2008;74(1):22–33.
